# Cerebral oxygen extraction across different exercise intensities: Role of arterial $P_{CO_{2}}$

**DOI:** 10.1113/EP092724

**Published:** 2025-12-01

**Authors:** L. Madden Brewster, Travis Dylan Gibbons, Hannah Grace Caldwell, Connor A. Howe, Jennifer S. Duffy, Andrew R. Steele, Justin A. Monteleone, Jay M. J. R. Carr, Jodie Lauren Koep, Tenasia D. R. Monaghan, David B. MacLeod, Philip N. Ainslie

**Affiliations:** ^1^ Centre for Heart, Lung and Vascular Health, School of Health and Exercise Sciences University of British Columbia Kelowna British Columbia Canada; ^2^ Department of Biological Sciences, College of the Environment, Forestry, and Natural Sciences Northern Arizona University Flagstaff Arizona USA; ^3^ The August Krogh Section for Human Physiology, Department of Nutrition, Exercise and Sports University of Copenhagen Copenhagen Denmark; ^4^ Department of Anesthesiology Duke University Medical Center Durham North Carolina USA

**Keywords:** cerebral, exercise, oxygen extraction

## Abstract

Stability in cerebral oxygen extraction fraction (OEF) is typically determined by alterations in cerebral blood flow (CBF). At rest, arterial partial pressure of carbon dioxide ($P_{\mathrm{aC}O_{2}}$) and OEF exhibit a strong inverse relationship owing to the powerful influence of $P_{\mathrm{aC}O_{2}}$ on cerebral resistance, CBF and therefore oxygen delivery; however, it is unclear whether this relationship also exists during exercise, especially when supramaximal, during which marked hyperventilation‐induced reductions in $P_{\mathrm{aC}O_{2}}$ induce cerebrovascular vasoconstriction and lower CBF. We determined whether: (1) supramaximal exercise yields the largest change in OEF versus lower intensities, correlated with reductions in $P_{\mathrm{aC}O_{2}}$; and (2) declines in $P_{\mathrm{aC}O_{2}}$ (independent of exercise) determine changes in OEF. Blood was sampled from the brachial/radial artery and internal jugular vein during: (1) 60 min, 34% maximal O_2_ uptake (SUB; *n* = six males, six females); (2) 4 min, 90% maximal O_2_ uptake (MAX; *n* = six males, six females); (3) 1−2 min of high‐intensity sprinting, ∼110% maximal O_2_ uptake (HIS; *n* = six males, five females); and (4) resting hyperventilation‐induced hypocapnia (HYPO; *n* = six males, five females). OEF was calculated as: [$\left(\mathrm{arterial}\;O_{2}\;\mathrm{content}-\mathrm{jugular}\;\mathrm{venous}\;O_{2}\;\mathrm{content}\right)/\mathrm{arterial}\;O_{2}\;\mathrm{content}]\;\times \;100.$ The ΔOEF was greatest during HIS [estimated marginal mean: 15.6% (95% confidence interval: 12.4, 18.8)] and HYPO [17.7% (14.5, 20.9)] compared with SUB [−0.9% (−4.0, 2.1); *p <* 0.0001 vs. HIS and vs. HYPO] and MAX [2.5% (−0.5, 5.6); *p <* 0.0001 vs. HIS and vs. HYPO]. Reductions in $\Delta P_{\mathrm{aC}O_{2}}$ were greatest in HIS [−12.9 mmHg (−14.6, −11.2)] and HYPO [−9.2 mmHg (−10.9, −7.6)] compared with MAX [−6.2 mmHg (−7.8, −4.6)] and SUB [1.4 mmHg (−0.2, 2.9); all comparisons *p <* 0.0001]. The ΔOEF was inversely related to $\Delta P_{\mathrm{aC}O_{2}}$ both in the pooled analysis [β = −1.65 (−2.23, −1.07); *p <* 0.0001] and within each of the conditions. In conclusion, probably owing to reductions in CBF, hypocapnia per se increased OEF in a similar manner to supramaximal sprinting, indicating that exercise is non‐obligatory in this process.

## Abstract (highlights)

- **What is the central question of this study?**
  We aimed to determine how cerebral oxygen extraction fraction (OEF) was altered across a range of cycling intensities including supramaximal exercise and the role of arterial partial pressure of carbon dioxide ($P_{\mathrm{aC}O_{2}}$).
- **What is the main finding and its importance?**
  Supramaximal exercise induced the greatest reductions in $P_{\mathrm{aC}O_{2}}$ and elevations in OEF compared with submaximal and maximal exercise. Inducing hypocapnia per se at rest increased OEF in a similar manner to supramaximal sprinting, indicating that exercise is non‐obligatory in this process. The $P_{\mathrm{aC}O_{2}}$ showed consistent associations with OEF across all conditions, supporting hypocapnia as a key mechanism to extract oxygen in the human brain, independent of exercise.

## 1 INTRODUCTION

The brain exhibits exceptional metabolic activity, consuming 15%−25% of the glucose and oxygen available to the body at rest, despite its relatively small size (Clarke & Sokoloff, 1999). Limited by its substrate and oxygen storage capacity (Oz et al., 2007), the brain must rely on the tight orchestration of oxygen delivery and extraction to support cerebral metabolic demand. Paradoxically, however, the brain has a 3‐fold lower cerebral oxygen extraction fraction (OEF) than other organs with high energy requirements, such as the heart and muscle during exercise (Lassen, 1959; Skattebo et al., 2020). The OEF is typically defined as the difference in arterial and venous oxygen content, to reflect the fraction of oxygen extracted by tissue as blood passes through the capillary bed. In the brain, the OEF serves as an index for metabolic activity, which aids in the regulation of cerebral blood flow (CBF) and cerebral blood volume to meet the demands of cerebral metabolism (reviewed by Engle et al., 2024; Jiang & Lu, 2022). During progressive exercise to maximal capacity, the cerebral delivery of oxygen (CDO_2_) remains remarkably stable, owing to appropriate changes in cerebral blood flow (CBF) and arterial oxygen content ($C_{aO_{2}}$); therefore, there is little need or stimulus for OEF to change (Smith & Ainslie, 2017). In contrast, supramaximal workloads elicit hyperventilation and, as such, marked reductions in arterial partial pressure of carbon dioxide ($P_{\mathrm{aC}O_{2}}$) occur (Martin‐Rincon et al., 2021). Such hypocapnia, via its powerful influence on cerebral vasoconstriction (Caldwell et al., 2024; Howe et al., 2020), leads to marked reductions in CBF, hence CDO_2_, therefore provoking a metabolic stimulus to increase OEF. For example, at least at rest, it has been reported in otherwise healthy volunteers, probably owing to reductions in CBF, that OEF is inversely and highly correlated with end‐tidal or arterial $P_{CO_{2}}$ (Caldwell et al., 2024; Jiang, Lin, Liu, Sur, Xu, Hazel, Pottanat, Yasar et al., 2020).

It is unclear whether the relationship between $P_{\mathrm{aC}O_{2}}$ and OEF also exists during exercise, especially supramaximal exercise, during which marked changes in $P_{\mathrm{aC}O_{2}}$ occur in conjunction with increased brain activity (Dalsgaard et al., 2004). In an earlier study that quantified the brain carbon uptake during cerebral activation (with and without β‐blockade in eight males), arterial‐to‐internal jugular venous oxygen tension differences were measured during moderate‐ and maximal‐intensity exercise (Dalsgaard et al., 2004); however, the oxygen content difference, which includes changes in haemoglobin (which increases following exercise), and therefore OEF, was not calculated; blood gases were not corrected for changes in body temperature; and the potential direct and indirect roles of $P_{\mathrm{aC}O_{2}}$ in mediating changes in OEF were not discussed. As such, although OEF is a key physiological index of the homeostasis of oxygen demand and supply in the human brain that is altered in many pathologies (reviewed by Engle et al., 2024; Jiang & Lu, 2022), comparison of OEF between different intensities of exercise has not been reported previously.

Therefore, we sought to determine whether OEF differs across a range of cycling exercise intensities, including supramaximal high‐intensity exercise. Furthermore, to isolate the role of $P_{\mathrm{aC}O_{2}}$ per se, we examined the relationship between OEF and $P_{\mathrm{aC}O_{2}}$ independent of exercise. We aimed to explore: (1) whether supramaximal exercise would yield the largest change in OEF compared with lower intensities and whether this would be correlated with reductions in $P_{\mathrm{aC}O_{2}}$; and (2) whether declines in $P_{\mathrm{aC}O_{2}}$ per se (independent of exercise) would determine these changes in OEF. To explore these *post hoc* aims, in 23 healthy volunteers, we used an invasive and gold‐standard transcerebral blood‐sampling model that was conducted over three separate experimental protocols using various exercise intensities. In a final set of conditions, we explored the role of hypocapnia per se independent of exercise.

## 2 MATERIALS AND METHODS

### 2.1 Ethical approval

Ethical approval was granted by the Clinical Research Ethics Board at the University of British Columbia Okanagan (H22‐01091, H23‐02303 and H21‐00773) and according to the principles set by the *Declaration of Helsinki* (clinical trial registration number: NCT06000605). All participants provided written informed consent prior to participating in the study.

### 2.2 Experimental protocol

The research questions addressed herein are hypothesis generating and involve *post hoc* analyses; as such, some of the data, specifically haematological variables, have been reported previously in separate contexts (Gibbons et al., 2024). However, the present experimental questions involve new analyses and aims that have not been reported previously. Three cycling protocols were compared: submaximal steady state (SUB); maximal steady state (MAX); and supramaximal high‐intensity sprinting (HIS). In separate trials, to determine the influence of $P_{\mathrm{aC}O_{2}}$ independent of exercise, a resting hyperventilation‐induced hypocapnia condition (HYPO) was also assessed, during which participants hyperventilated in a supine position until a steady‐state drop in $P_{\mathrm{aC}O_{2}}$ of ∼10 mmHg was achieved (∼2−3 min). The data from SUB and MAX were collected from the same study and include the same participants (*n* = 12). A few individuals participated in multiple study conditions: *n* = 1 volunteer participated in all four conditions, separated by ≥2 years; *n* = 1 individual participated in SUB/MAX and HYPO, separated by 3 years; *n* = 5 volunteers participated in both HYPO and HIS groups, separated by 5 years; and *n* = 3 participated in SUB/MAX and HIS, separated by 2 years. We believe that enough time passed between testing visits in these individuals, especially in view of the normal within‐ and between‐day individual variability, that inclusion as paired samples need not be considered essential in our statistics. Although all participants were healthy after screening, fitness status was not assessed in all studies included in the present analysis; however, on an individual level, fitness would not be expected to have an impact on the within‐individual OEF–$P_{\mathrm{aC}O_{2}}$ relationships. Given the extended time between conditions, individuals are treated as independent samples across conditions. Nevertheless, in the case where *post hoc* statistical testing was conducted, differences between SUB and MAX were also confirmed using paired testing.

All participants completed a maximal aerobic power test on a separate day prior to testing to determine appropriate work rates for ensuing exercise protocols. On the day of experimentation, participants were instrumented with general‐purpose sterile thermistors (RET‐1, Physitemp Instruments, Clifton, NJ, USA) inserted via the rectum (depth of ∼15 cm) to correct blood gases for core temperature. Thereafter, participants were cannulated with a radial or brachial artery and internal jugular venous catheter (see section 2.4) to allow for simultaneous arteriovenous blood collection. Blood was sampled simultaneously from both arterial and jugular venous lines at rest (PRE) and at the end of exercise or closely thereafter (POST), the details of which are described, for each of the conditions, below. Participants avoided vigorous exercise and alcohol for ≥12 h prior to testing. Participants had a light meal and ingested caffeine according to their normal routine up to 2 h before testing.

### 2.3 Participants

Healthy males and females participated in either SUB and MAX (six females and six males), HIS (five females and six males) or HYPO (five females and six males) conditions. All participants were normotensive, normal weight, non‐smokers and otherwise healthy, with no previous history of cardiovascular, respiratory, metabolic or cerebrovascular disease. There were no differences in age, weight or body mass index between groups (Table 1).

**TABLE 1:** Subject characteristics.

CONDITION	*n*	Age (years)	Weight (kg)	Body mass index (kg/m^2^)
SUB AND MAX	6F/6M	28.2 ± 4.6	71.6 ± 10.1	23.2 ± 2.3
HIS	5F/6M	28.0 ± 3.7	67.8 ± 9.2	22.4 ± 1.7
HYPO	5F/6M	26.5 ± 3.8	70.3 ± 8.0	23.1 ± 1.9

*Note*: All data are presented as mean ± sd. Abbreviations: F, female; M, male; HIS, high intensity sprinting; HYPO, hypocapnia (resting); MAX, maximal exercise; SUB, submaximal exercise.

### 2.4 Arterial and venous catheterization

Using sterile technique, a 20 gauge arterial catheter (Arrow, Markham, ON, Canada) was advanced into either the radial or the brachial artery under local anaesthesia (lignocaine, 1.0%) with ultrasound guidance. A 13 gauge central venous catheter (Cook Medical, Bloomington, IN, USA) was inserted into the right internal jugular vein and advanced 15 cm cephalad in sterile conditions and local anaesthesia with ultrasound guidance (Schell & Cole, 2000). This technique, successfully performed by our group, has previously been demonstrated to lead to catheter tip placement in the jugular bulb, superior to the facial vein (Ainslie et al., 2014); details have been described previously elsewhere (Ainslie et al., 2014; Schell & Cole, 2000). Fulfilment of these techniques leads to ≤3% contamination by extracerebral blood (Schell & Cole, 2000). Catheters were attached to in‐line wasteless blood sampling systems for repeated transcerebral blood sampling (Edwards Lifesciences, TruWave VAMP, CA, USA).

### 2.5 Blood sampling

Using preheparinized syringes (SafePICO, Radiometer, Copenhagen, Denmark), ∼1.0 mL samples of radial arterial and internal jugular venous blood were obtained simultaneously and analysed immediately thereafter using a commercial blood gas analyser (ABL90 FLEX, Radiometer) at each experimental stage. Samples were analysed for the partial pressures of arterial and venous carbon dioxide ($P_{\mathrm{aC}O_{2}}$ and $P_{\mathrm{vC}O_{2}}$) and oxygen ($P_{aO_{2}}$ and $P_{vO_{2}}$), oxygen saturation ($S_{aO_{2}}$ and $S_{vO_{2}}$), oxygen, glucose and lactate content ($C_{aO_{2}}$, $C_{vO_{2}}$, Glc_a_, Glc_v_, Lac_a/v_ and Lac_a/v_, respectively), calculated haemoglobin concentration ([Hb]) and pH.

### 2.6 Maximal aerobic exercise testing

All participants completed a progressive maximal aerobic exercise test on a cycle ergometer within 1 week of the testing period to determine appropriate work rates for the experimental conditions. Participants were instrumented with a fitted mask connected to a metabolic cart (Vyntus CPX, Vyaire Medical or Quark CPET, Cosmed) to measure oxygen consumption (${\dot{V}}_{O_{2}}$). For the HIS protocol, all participants began with a 1 min resting period, and all tests began at 100 W, with progressive 25 W increases every 1 min (SUB/MAX) or 3 min (HIS) until volitional exhaustion. Work rate percentages were calculated using the linear regression equation generated from the work rate of the corresponding steady‐state ${\dot{V}}_{O_{2}}$ average for each stage.

### 2.7 Exercise protocols

#### 2.7.1 Submaximal and maximal exercise protocol

The same cohort participated in both the SUB and MAX exercise conditions, which were part of a larger study protocol. Six females and six males were tested (*n* = 12). Participants began by resting in a semi‐recumbent position for ≥5 min prior to beginning exercise. Blood samples from the radial artery and internal jugular vein were collected simultaneously in the last minute prior to exercise commencement and immediately analysed with a blood gas analyser as described above. Once baseline data collection was complete, participants were instructed to begin exercising on a semi‐recumbent cycle ergometer. Their work rate was set to elicit 34% of maximal oxygen consumption (${\dot{V}}_{O_{2}\max}$) for 60 min (SUB). During the last 15 s of the SUB exercise trial, arterial and jugular venous blood was sampled simultaneously. After a 15 min rest period, baseline measurements for the MAX conditions were taken. Thereafter, participants completed an exercise protocol whereby three incremental exercise stages of 30%, 60% and 90% of ${\dot{V}}_{O_{2}\max}$ for 4 min each were completed. Blood samples for the post MAX conditions were collected in the last 15 s of exercise in the final 90% ${\dot{V}}_{O_{2}\max}$ stage.

#### 2.7.2 High‐intensity sprinting exercise protocol

For the HIS conditions, six males and five females were tested (*n* = 11). These conditions were similar to those outlined above; blood was collected and analysed immediately from the radial artery and internal jugular vein in the last minute prior to exercise commencement from a semi‐recumbent resting position. Upon completion of baseline measurements, participants began the exercise protocol, which consisted of exercising at a work rate of ∼110% of ${\dot{V}}_{O_{2}\max}$ for ∼60 s, followed by a recovery period of 1−2 min. This was repeated one more time before a final round of high‐intensity sprinting at ∼110% of ${\dot{V}}_{O_{2}\max}$ for as long as possible (i.e., until volitional exhaustion, ∼1−2 min; HIS). Approximately 2−3 min after completion of the third round of supramaximal exercise (HIS), arterial and venous blood were sampled concurrently. HIS was used as an exercise condition in the present study owing to its marked influence on ventilation and thus $P_{\mathrm{aC}O_{2}}$.

#### 2.7.3 Resting hypocapnia conditions

Participants lay supine, instrumented with a mouthpiece connected to a pneumotach (1000 L, ADInstruments, Dunedin, NZ) and gas analyser (model ML206, ADInstruments, Dunedin, NZ). Following 15 min of supine rest, individuals were instructed to hyperventilate at their desired combination of frequency and tidal volume to induce a steady‐state (>5 min) level of hypocapnia, a ∼10 mmHg decrease from baseline, during which end‐tidal $P_{O_{2}}$ was clamped at baseline using a dynamic end‐tidal forcing system described previously (Tymko et al., 2015, 2016). Blood samples were taken in the last 30 s of hyperventilation after ∼5 min of steady state of the desired reduction in partial pressure of end‐tidal CO_2_ was achieved.

#### 2.7.4 Calculations

All blood gases were corrected for changes in core temperature determined from calibrated rectal thermistor using previously derived constants and logarithmic equations (Severinghaus, 1966). Arterial ($C_{aO_{2}}$) and venous ($C_{vO_{2}}$) oxygen contents were determined using the following:

$$C_{aO_{2}}\left(\mathrm{mL}/\mathrm{dL}\right)=\left[1.36\times \mathrm{Hb}\;\left(g/\mathrm{dL}\right)\times S_{a}O_{2}\left(\%\right)\right]+0.0003\times P_{aO_{2}}\left(\mathrm{mmHg}\right)$$

$$C_{vO_{2}}\left(\mathrm{mL}/\mathrm{dL}\right)=\left[1.36\times \mathrm{Hb}\;\left(g/\mathrm{dL}\right)\times S_{v}O_{2}\left(\%\right)\right]+0.0003\times P_{vO_{2}}\left(\mathrm{mmHg}\right)$$

where 1.36 represents the binding capacity of Hb for a given $S_{O_{2}}$, and 0.003 is the percentage of O_2_ dissolved in the blood (Lumb & Thomas, 2020; West & Luks, 2020).

The difference between $C_{aO_{2}}$ and $C_{vO_{2}}$ was used to determine cerebral OEF as follows:

$$\mathrm{OEF}\;\left(\%\right)=\frac{C_{aO_{2}}\left(\mathrm{mL}/\mathrm{dL}\right)-C_{vO_{2}}\left(\mathrm{mL}/\mathrm{dL}\right)}{C_{aO_{2}}\left(\mathrm{mL}/\mathrm{dL}\right)}\times 100$$

### 2.8 Statistical analyses

Data are presented as the mean ± SD or estimated marginal mean [95% confidence intervals (CIs)]. Statistical analyses were performed and figures generated with RStudio software (The R Foundation for Statistical Computing, R v.4.3.3, 2024). Pre‐ and postexercise comparisons and within‐subject arterial and venous data were analysed by Student's paired *t*‐test. Linear mixed‐effects models (‘lme4’ package, v.1.1.37) were used to assess the differences between conditions (SUB, MAX, HIS and HYPO; fixed effect), accounting for repeated measures by subject (random intercept). Model assumptions were evaluated by visual inspection of histograms and *Q–Q* plots (for normality) and plots of residual versus fitted values (for homoscedascity). To examine the relationships between ΔOEF and $\Delta P_{\mathrm{aC}O_{2}}$ and between ΔOEF and ΔpH_Arterial_, we fitted linear mixed‐effects models, including the predictor ($\Delta P_{\mathrm{aC}O_{2}}$ and ΔpH_Arterial_), condition (SUB, MAX, HIS and HYPO), their interaction, and a random intercept for participant. Regression lines (in Figure 2a, b) were calculated by averaging fixed‐effect predictions equally across conditions, yielding a marginal (pooled) line with corresponding pooled slope and 95% CIs. Condition‐specific regression lines (in Figure 2c, d) were plotted based on the fixed‐effects predictions of the interaction model. Type III ANOVA (Wald χ^2^ tests) was used as the omnibus test of fixed effects, including the interaction terms, to assess whether slopes differed between conditions. Within‐condition simple slopes (slope estimate, 95% CI, *p*‐value) and pairwise slope differences with Tukey's adjustment for multiple comparisons were obtained using estimated marginal trends (‘emmeans’ package v.1.11.2). Statistical significance was set at *p <* 0.05 for all statistical tests.

## 3 RESULTS

### 3.1 Blood gases, oximetry and metabolites across exercise

Participant characteristics are presented in Table 1. Participants were generally young and healthy, with similar anthropometric characteristics between groups. Arterial and jugular venous blood gases, oximetry and OEF data pre‐ and post‐conditions are presented in Table 2 as the mean ± SD, with corresponding *p*‐values in Supplementary Materials 1). The $\Delta P_{\mathrm{aC}O_{2}}$ was reduced from PRE to POST across MAX (39.2 ± 3.4 vs. 33.0 ± 3.9 mmHg, *p <* 0.0001), HIS (40.5 ± 2.7 vs. 27.6 ± 3.1 mmHg, *p <* 0.0001) and HYPO (44.0 ± 0.9 vs. 34.8 ± 2.1 mmHg, *p <* 0.0001) and increased across SUB (38.7 ± 3.7 vs. 40.1 ± 3.3 mmHg, *p =* 0.0071). The change in arterial pH for HYPO from PRE to POST was positive (PRE vs. POST: 7.38 ± 0.01 vs. 7.45 ± 0.01, *p <* 0.0001; i.e., respiratory alkalosis), whereas MAX (PRE vs. POST: 7.42 ± 0.01 vs. 7.28 ± 0.04, *p <* 0.0001) and HIS (PRE vs. POST: 7.52 ± 0.18 vs. 7.24 ± 0.15, *p <* 0.0001) decreased from PRE to POST exercise (i.e., acidosis). Arterial pH was unchanged across exercise in the SUB condition (PRE vs. POST: 7.42 ± 0.01 vs. 7.41 ± 0.01, *p =* 0.1196). Although much smaller than the decrease from baseline in arterial [HCO_3_^−^] in MAX (PRE vs. POST: 24.7 ± 2.3 vs. 15.1 ± 2.4 mmol/L, *p <* 0.0001) and HIS (PRE vs. POST: 28.5 ± 2.9 vs. 10.3 ± 2.4 mmol/L, *p <* 0.0001), there was also a modest decline in [HCO_3_^−^] for HYPO (PRE vs. POST: 25.5 ± 1.0 vs. 24.1 ± 1.2 mmol/L, *p <* 0.0001). There was no effect of SUB exercise on arterial [HCO_3_^−^] (PRE vs. POST: 24.8 ± 2.2 vs. 25.2 ± 2.2  mmol/L, *p =* 0.1583). The $C_{aO_{2}}$ was increased from PRE to POST exercise in SUB (18.7 ± 1.4 vs. 19.1 ± 1.5, *p =* 0.0113), MAX (19.1 ± 1.4 vs. 20.6 ± 1.5, *p <* 0.0001) and HIS (18.2 ± 1.6 vs. 20.0 ± 1.8, *p <* 0.0001), but not the resting HYPO (18.0 ± 1.1 vs. 18.2 ± 1.3, *p =* 0.2762) conditions.

**TABLE 2:** Blood gases, oximetry and metabolites.

Parameter	SUB		MAX		HIS		HYPO	
	PRE	POST	PRE	POST	PRE	POST	PRE	POST
$P_{O_{2}}$, mmHg								
Arterial	97.9 ± 5.6†	97.2 ± 3.5†	98.4 ± 4.8†	100.3 ± 9.2†	88.3 ± 9.6†	115.3 ± 5.1†, *	88.1 ± 7.0†	89.5 ± 6.4†
Venous	32.2 ± 4.2†	33.2 ± 3.0†	31.8 ± 2.6†	34.5 ± 4.7†, *	35.6 ± 1.8†	35.9 ± 2.8†	40.0 ± 2.9†	29.1 ± 1.9†, *
Ateriovenous difference	65.7 ± 8.0	64.0 ± 5.4	66.6 ± 5.0	65.7 ± 11.8	52.7 ± 9.7	79.3 ± 6.8*	48.0 ± 7.8	60.3 ± 6.7*
$P_{CO_{2}}$, mmHg								
Arterial	38.7 ± 3.7†	40.1 ± 3.3†, *	39.2 ± 3.4†	33.0 ± 3.9†, *	40.5 ± 2.7†	27.6 ± 3.1†, *	44.0 ± 0.9†	34.8 ± 2.1†, *
Venous	50.1 ± 3.9†	52.2 ± 3.7†, *	50.9 ± 3.7†	48.6 ± 3.8†, *	49.6 ± 2.2†	43.7 ± 1.9†, *	50.9 ± 3.8†	46.9 ± 5.0†, *
Arteriovenous difference	−11.4 ± 3.0	−12.1 ± 2.6	−11.8 ± 2.2	−15.6 ± 2.7*	−9.1 ± 1.3	−16.2 ± 3.0*	−6.8 ± 3.8	−12.1 ± 3.8*
pH								
Arterial	7.42 ± 0.02†	7.41 ± 0.01†	7.42 ± 0.01†	7.28 ± 0.04†, *	7.52 ± 0.18†	7.24 ± 0.15†, *	7.38 ± 0.01†	7.45 ± 0.01†, *
Venous	7.36 ± 0.01†	7.35 ± 0.01†	7.36 ± 0.01†	7.23 ± 0.03†, *	7.47 ± 0.17†	7.20 ± 0.14†, *	7.35 ± 0.01†	7.38 ± 0.01†, *
Arteriovenous difference	0.06 ± 0.02	0.06 ± 0.01	0.06 ± 0.01	0.05 ± 0.01*	0.05 ± 0.01	0.04 ± 0.01	0.03 ± 0.02	0.07 ± 0.01*
HCO_3_^−^, mmol/L								
Arterial	24.8 ± 2.2†	25.2 ± 2.2†	24.7 ± 2.3†	15.1 ± 2.4†, *	28.5 ± 2.9†	10.3 ± 2.4†, *	25.5 ± 1.0†	24.1 ± 1.2†, *
Venous	27.3 ± 2.6†	27.8 ± 2.3†	27.4 ± 2.2†	19.3 ± 2.6†, *	31.0 ± 3.5†	14.7 ± 2.8†, *	27.3 ± 1.8†	27.4 ± 2.6†
Arteriovenous difference	−2.5 ± 1.2	−2.6 ± 0.9	−2.7 ± 0.7	−4.3 ± 1.0*	−2.5 ± 0.8	−4.4 ± 1.1*	−1.8 ± 1.1	−3.4 ± 2.1*
$S_{O_{2}}$, %								
Arterial	98.2 ± 0.6†	97.9 ± 0.7†	98.1 ± 0.5†	97.0 ± 1.3†, *	97.7 ± 1.3†	97.9 ± 0.5†	95.3 ± 0.9†	96.2 ± 0.5†, *
Venous	57.7 ± 8.1†	57.7 ± 6.1†	55.9 ± 5.5†	52.5 ± 6.1†, *	67.2 ± 3.7†	51.8 ± 4.2†, *	71.1 ± 3.5†	54.6 ± 3.6†, *
Arteriovenous difference	40.6 ± 8.1	40.2 ± 6.2	42.3 ± 5.6	44.6 ± 7.0	30.5 ± 4.1	46.1 ± 4.4*	24.2 ± 3.6	41.6 ± 3.6*
Haematocrit, %								
Arterial	43.0 ± 3.3	44.0 ± 3.5*	43.8 ± 3.4	47.8 ± 3.6*	42.2 ± 3.5	45.8 ± 4.1*	42.6 ± 2.6	42.6 ± 3.1
Venous	42.7 ± 3.2	43.9 ± 3.5*	43.7 ± 3.3	47.6 ± 3.9*	42.2 ± 3.5	46.1 ± 4.3*	43.0 ± 3.0	42.2 ± 3.5
Arteriovenous difference	0.3 ± 1.2	0.1 ± 0.7	0.1 ± 0.8	0.2 ± 0.6	−0.1 ± 0.4	−0.2 ± 0.4	−0.3 ± 0.8	0.4 ± 3.4
Haemoglobin, g/dL								
Arterial	14.0 ± 1.1	14.3 ± 1.2*	14.3 ± 1.1	15.6 ± 1.2*	13.7 ± 1.1	15.0 ± 1.3*	13.9 ± 0.9	13.9 ± 1.0
Venous	13.9 ± 1.1	14.3 ± 1.1*	14.3 ± 1.1	15.6 ± 1.2*	13.8 ± 1.1	15.0 ± 1.4*	14.0 ± 1.0	14.1 ± 1.0
Arteriovenous difference	0.1 ± 0.4	0.0 ± 0.2	0.0 ± 0.3	0.1 ± 0.2	−0.0 ± 0.1	−0.1 ± 0.1	−0.1 ± 0.3	−0.2 ± 0.2
Glucose, mmol/L								
Arterial	6.8 ± 1.1†	5.2 ± 0.5†, *	5.5 ± 0.6†	5.5 ± 0.5†	5.3 ± 0.3†	6.4 ± 0.6†, *	5.3 ± 0.4†	5.4 ± 0.4†
Venous	6.1 ± 1.1†	4.5 ± 0.5†, *	4.8 ± 0.6†	4.7 ± 0.6†	4.8 ± 0.3†	5.7 ± 0.6†, *	4.9 ± 0.3†	4.6 ± 0.6†, *
Arteriovenous difference	0.7 ± 0.2	0.7 ± 0.1	0.7 ± 0.1	0.8 ± 0.1	0.5 ± 0.1	0.8 ± 0.1*	0.4 ± 0.1	0.7 ± 0.4*
Lactate, mmol/L								
Arterial	1.1 ± 0.4	0.7 ± 0.2†, *	0.7 ± 0.2	13.1 ± 1.5†, *	0.8 ± 0.2	19.5 ± 4.2†, *	0.5 ± 0.2†	0.5 ± 0.2†
Venous	1.1 ± 0.4	0.7 ± 0.2†, *	0.8 ± 0.2	11.6 ± 1.2†, *	0.8 ± 0.1	18.0 ± 4.1†, *	0.5 ± 0.2†	0.6 ± 0.2†
Arteriovenous difference	−0.0 ± 0.1	−0.1 ± 0.0*	−0.1 ± 0.0	1.4 ± 0.4*	−0.0 ± 0.1	1.5 ± 0.5*	−0.1 ± 0.1	−0.1 ± 0.1
O_2_ content								
Arterial	18.7 ± 1.4†	19.1 ± 1.5†, *	19.1 ± 1.4†	20.6 ± 1.5†, *	18.2 ± 1.6†	20.0 ± 1.8†, *	18.0 ± 1.1†	18.2 ± 1.3†
Venous	10.9 ± 1.7†	11.2 ± 1.6†	10.8 ± 1.3†	11.0 ± 1.5†	12.5 ± 1.5†	10.6 ± 1.4†, *	13.5 ± 1.1†	10.4 ± 1.0†, *
Arteriovenous difference	7.9 ± 1.5	7.9 ± 1.2	8.3 ± 1.1	9.6 ± 1.5*	5.7 ± 0.7	9.4 ± 1.1*	4.6 ± 0.8	7.8 ± 0.9*
OEF, %								
	42.0 ± 8.0	41.5 ± 6.3	43.6 ± 5.5	46.5 ± 6.4	31.4 ± 3.8	47.3 ± 4.3*	25.3 ± 4.0	42.9 ± 3.8*

*Note*: Data are presented as the mean ± SD. Abbreviations: HCO_3_^−^, bicarbonate; HIS, high‐intensity sprinting; HYPO, hypocapnia (resting); OEF, cerebral oxygen extraction fraction; MAX, maximal exercise; $P_{CO_{2}}$, partial pressure of carbon dioxide; $P_{O_{2}}$, partial pressure of oxygen; $S_{O_{2}}$, oxygen saturation; SUB, submaximal exercise.

^*^ Different from PRE (*p <* 0.05).

^†^ Different from arterial (*p <* 0.05).

### 3.2 Blood gases, oximetry and metabolites between conditions

The change in OEF (ΔOEF), arterial pH (ΔpH_Arterial_) and $\Delta P_{\mathrm{aC}O_{2}}$ (Δ$\Delta P_{\mathrm{aC}O_{2}}$) from baseline compared between conditions are presented in Figure 1 as the estimated marginal mean (95% CI). The change in all blood gases, oximetry and metabolites from baseline between each of the conditions are presented in Table 3, with corresponding pairwise comparisons in Supplementary Materials 2. Across exercise, the $\Delta P_{\mathrm{aC}O_{2}}$ was greatest in HIS compared with MAX [estimated marginal mean (95% CI); HIS vs. MAX: −12.88 (−14.56, −11.21) vs. −6.22 (−7.8, −4.63) mmHg, *p <* 0.0001] and SUB [HIS vs. SUB: −12.88 (−14.56, −11.21) vs. 1.35 (−0.23, 2.94) mmHg, *p <* 0.0001]. The $\Delta P_{\mathrm{aC}O_{2}}$ was greater for MAX compared with SUB [MAX vs. SUB: −6.22 (−7.8, −4.63) vs. 1.35 (−0.23, 2.94) mmHg, *p <* 0.0001]. The $\Delta P_{\mathrm{aC}O_{2}}$ for HYPO also differed significantly from SUB [HYPO vs. SUB: −9.23 (−10.89, −7.56) vs. 1.35 (−0.23, 2.94) mmHg, *p <* 0.0001] and HIS [HYPO vs. HIS: −9.23 (−10.89, −7.56) vs. −12.88 (−14.56, −11.21) mmHg, *p =* 0.0179] only. The ΔpH_Arterial_ was significantly different between all groups [SUB: −0.01 (−0.03, 0.01), MAX: −0.14 (−0.16, −0.12), HIS: −0.28 (−0.3, −0.26), HYPO: 0.07 (0.05, 0.1), all pairwise *p <* 0.0001]. The ΔOEF was significantly greater in HIS and HYPO compared with SUB [HIS vs. SUB: 15.61 (12.44, 18.77) vs. −0.94 (−4.0, 2.12), *p <* 0.0001; HYPO vs. SUB: 17.69 (14.51, 20.87) vs. −0.94 (−4.0, 2.12), *p <* 0.0001] and MAX [HIS vs. MAX: 15.61 (12.44, 18.77) vs. 2.52 (−0.54, 5.58), *p <* 0.0001; HYPO vs. MAX: 17.69 (14.51, 20.87) vs. 2.52 (−0.54, 5.58), *p <* 0.0001]. The ΔOEF did not differ between HIS and HYPO (*p =* 0.733) or between SUB and MAX (*p =* 0.24).

**FIGURE 1:**
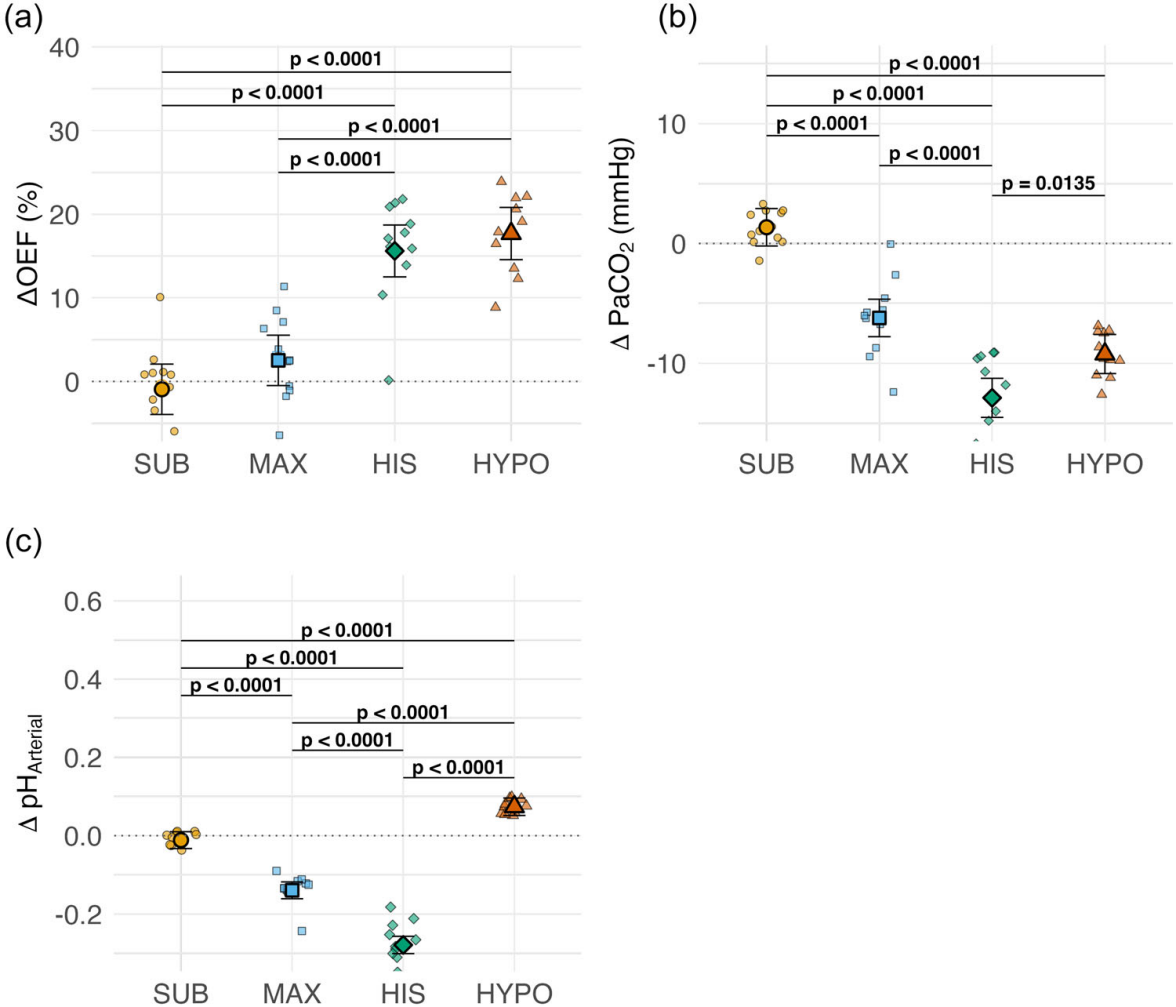
The change in cerebral oxygen extraction (ΔOEF; a), arterial $P_{CO_{2}}$ ($\Delta P_{\mathrm{aC}O_{2}}$; b) and arterial pH (ΔpH_Arterial_; c) from baseline in each of the conditions. All data are presented as individual data as smaller symbols, with estimated marginal means represented by large symbols and 95% confidence interval. Number of participants per condition: SUB, *n* = 12; MAX, *n* = 12; HIS, *n* = 11; and HYPO, *n* = 11. Abbreviations: HIS, high intensity sprinting; HYPO, hypocapnia (resting); MAX, maximal exercise; SUB, submaximal exercise.

**TABLE 3:** The estimated marginal mean difference in blood gases, oximetry and metabolites from baseline in each of the conditions.

Variable	SUB (*n* = 12)	MAX (*n* = 12)	HIS (*n* = 11)	HYPO (*n* = 11)
$\Delta P_{aO_{2}}$, mmHg	−0.86 [−5.7, 3.99]^a^	1.72 [−3.13, 6.56]^a^	26.93 [21.81, 32.04]^b^	1.3 [−3.79, 6.38]^a^
$\Delta P_{vO_{2}}$, mmHg	1.17 [−0.69, 3.03]^a^	2.93 [1.07, 4.79]^a^	0.46 [−1.48, 2.39]^a^	−10.84 [−12.77, −8.9]^b^
$\Delta P_{\mathrm{aC}O_{2}}$, mmHg	1.35 [−0.23, 2.94]^a^	−6.22 [−7.8, −4.63]^b^	−12.88 [−14.56, −11.21]^c^	−9.23 [−10.89, −7.56]^b^
$\Delta P_{\mathrm{vC}O_{2}}$, mmHg	2.12 [1.11, 3.14]^a^	−2.37 [−3.39, −1.36]^b^	−5.85 [−6.93, −4.78]^c^	−3.92 [−4.99, −2.85]^bc^
ΔpH_a_	−0.01 −0.[−0.03, 0.01]^a^	−0.14 −0.[−0.16, −0.12]^b^	−0.28 −0.[−0.3, −0.26]^c^	0.07 [0.05, 0.1]^d^
ΔpH_v_	−0.01 −0.[−0.02, 0.01]^a^	−0.12 −0.[−0.14, −0.11]^b^	−0.27 −0.[−0.29, −0.25]^c^	0.04 [0.01, 0.06]^d^
ΔHCO_3_^−^_a_, mmol/L	0.43 −0.[−0.35, 1.2]^a^	−9.61 [−10.38, −8.83]^b^	−18.26 [−19.08, −17.44]^c^	−1.42 [−2.24, −0.61]^d^
ΔHCO_3_^−^_v_, mmol/L	0.54 −0.[−0.31, 1.4]^a^	−8.03 [−8.89, −7.17]^b^	−16.32 [−17.22, −15.41]^c^	0.11 −0.[−0.8, 1.01]^a^
$\Delta S_{aO_{2}}$, %	−0.35 −0.[−0.96, 0.25]^ab^	−1.12 [−1.73, −0.51]^a^	0.26 −0.[−0.38, 0.9]^bc^	0.89 [0.25, 1.53]^c^
$\Delta S_{vO_{2}}$, %	0.29 [−2.59, 3.16]^a^	−3.19 [−6.06, −0.32]^a^	−15.13 [−18.13, −12.13]^b^	−16.42 [−19.41, −13.42]^b^
ΔHct_a_, %	1.02 [0.42, 1.63]^a^	4.06 [3.46, 4.67]^b^	3.62 [2.98, 4.25]^b^	−0.02 −0.[−0.66, 0.61]^a^
ΔHct_v_,%	1.2 [0.14, 2.25]^a^	3.93 [2.87, 4.99]^b^	3.9 [2.81, 4.98]^b^	−0.79 [−1.89, 0.3]^c^
ΔHb_a_, g/dL	0.35 [0.15, 0.56]^a^	1.34 [1.13, 1.54]^b^	1.22 [1, 1.43]^b^	0.01 −0.[−0.21, 0.22]^a^
ΔHb_v_, g/dL	0.4 [0.19, 0.61]^a^	1.28 [1.08, 1.49]^b^	1.24 [1.02, 1.46]^b^	0.04 −0.[−0.18, 0.25]^a^
ΔGlucose_a_, mmol/L	−1.59 [−1.99, −1.18]^a^	−0.02 −0.[−0.42, 0.38]^b^	1.13 [0.71, 1.56]^c^	0.01 −0.[−0.41, 0.44]^b^
ΔGlucose_v_, mmol/L	−1.56 [−1.97, −1.15]^a^	−0.09 −0.[−0.5, 0.31]^b^	0.86 [0.43, 1.29]^c^	−0.3 −0.[−0.73, 0.12]^b^
ΔLactate_a_, mmol/L	−0.44 [−1.73, 0.86]^a^	12.38 [11.08, 13.67]^b^	18.79 [17.43, 20.16]^c^	−0.04 [−1.4, 1.32]^a^
ΔLactate_v_, mmol/L	−0.38 [−1.63, 0.86]^a^	10.84 [9.59, 12.08]^b^	17.23 [15.92, 18.55]^c^	0 [−1.31, 1.3]^a^
$\Delta C_{aO_{2}}$, mL/dL	0.38 [0.11, 0.65]^a^	1.52 [1.25, 1.78]^b^	1.74 [1.46, 2.03]^b^	0.17 −0.[−0.11, 0.45]^a^
$\Delta C_{vO_{2}}$, mL/dL	0.4 −0.[−0.17, 0.97]^a^	0.32 −0.[−0.26, 0.89]^a^	−1.92 [−2.52, −1.33]^b^	−3.07 [−3.66, −2.47]^c^
ΔOEF, %	−0.94 [−4, 2.12]^a^	2.52 −0.[−0.54, 5.58]^a^	15.61 [12.44, 18.77]^b^	17.69 [14.51, 20.87]^b^

*Note*: Data are presented as estimated marginal means [95% confidence interval]. Subscript ‘a’ represents brachial/radial arterial, and ‘v’ represents internal jugular venous data. Delta (Δ) refers to the difference between pre versus post conditions. Abbreviations: C_aO2_, arterial oxygen content; C_vO2_, venous oxygen content; Hct, haematocrit; Hb, haemoglobin; HCO_3_^−^, bicarbonate; HIS, high‐intensity sprinting; HYPO, hypocapnia (resting); MAX, maximal exercise; OEF, cerebral oxygen extraction fraction $P_{CO_{2}}$, partial pressure of carbon dioxide; $P_{O_{2}}$, partial pressure of oxygen; $S_{O_{2}}$, oxygen saturation; SUB, submaximal exercise. Statistically significant differences (*p <* 0.05) between conditions are represented by different letters.

### 3.3 Cerebral OEF is related to reductions in $\Delta P_{\mathrm{aC}O_{2}}$

Averaged equally across all conditions, a linear mixed‐effects model revealed a significant inverse association between ΔOEF and $\Delta P_{\mathrm{aC}O_{2}}$ [β = –1.65 (−2.23, −1.07), *p <* 0.0001], indicating that OEF increased by 1.65 percentage points per millimetre of mercury reduction in $\Delta P_{\mathrm{aC}O_{2}}$ (Figure 2a). Each condition (SUB, MAX, HIS and HYPO) demonstrated a similar association (Figure 2c), with the main effect of $\Delta P_{\mathrm{aC}O_{2}}$ significant (*p =* 0.0017) and no evidence of slope differences between conditions (interaction *p =* 0.272). When slopes were averaged equally across groups, the model estimated a positive association between ΔOEF and ΔpH_Arterial_ [β = 78.61 (7.83, 149.38), *p =* 0.030; Figure 2b], but the main effect of ΔpH_Arterial_ was not significant (*p =* 0.94). The ΔpH_Arterial_ × condition interaction showed a trend towards heterogeneity of slopes (*p =* 0.077), but did not reach significance. Condition‐specific slopes indicated significant positive relationships only in MAX [β = 86.71 (9.57, 163.85), *p =* 0.029] and HYPO [β = 206.9 (23.24, 390.57), *p =* 0.028; Figure 2d]. In contrast, there were no relationships in SUB (*P =* 0.941) and HIS (*P =* 0.571). Pairwise comparisons of all slopes revealed no significant differences after Tukey's adjustment (all adjusted *p *> 0.17).

**FIGURE 2:**
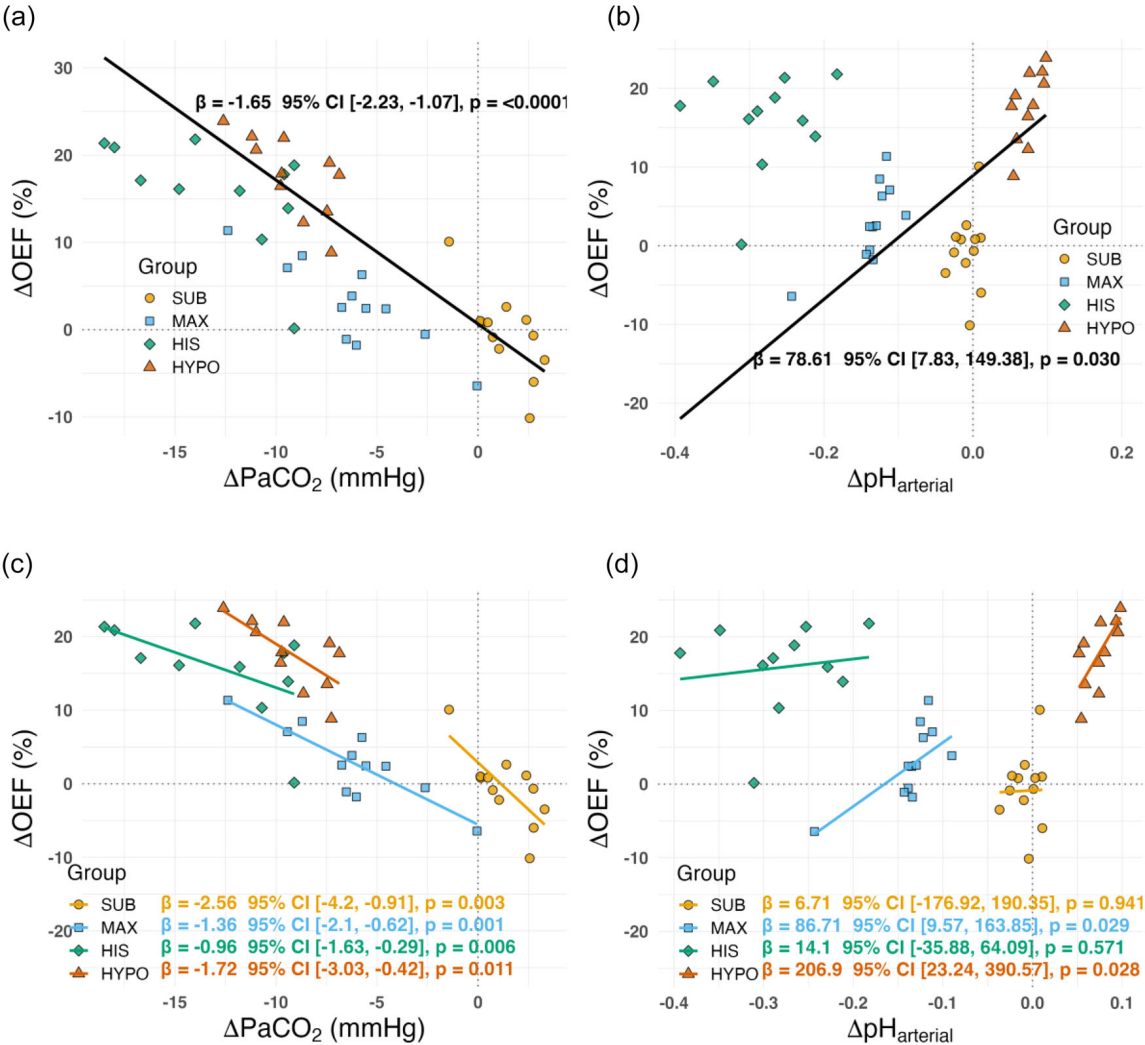
The absolute change in arterial $P_{CO_{2}}$ ($\Delta P_{\mathrm{aC}O_{2}}$; a) and arterial pH (ΔpH_Arterial_; b) versus the absolute change in cerebral oxygen extraction fraction (ΔOEF) shown as the pooled (marginal) regression line averaged equally across conditions. (c) Condition‐specific regression lines for $\Delta P_{\mathrm{aC}O_{2}}$ versus ΔOEF, with slope estimates from the mixed‐effects models annotated. Slopes were not significantly different between conditions (interaction *p* = 0.288). (d) Condition‐specific regression lines for ΔpH_Arterial_ versus ΔOEF, with slope estimates annotated. Slopes did not differ significantly between conditions (interaction *p* = 0.077). Number of participants per condition: SUB, *n* = 12; MAX, *n* = 12; HIS, *n* = 11; and HYPO, *n* = 11. Abbreviations: HIS, high‐intensity sprinting; HYPO, hypocapnia (resting); MAX, maximal exercise; SUB, submaximal exercise.

## 4 DISCUSSION

The primary new finding of the present study is that high‐intensity, supramaximal cycling induces a greater increase in OEF than that of other, less‐intense cycling exercise. Resting hyperventilation‐induced hypocapnia also yielded a similar increase in OEF to high‐intensity sprinting, probably owing to its influence on reducing CBF, indicating that changes in OEF occur independently of exercise. These results provide evidence that reduction in $\Delta P_{\mathrm{aC}O_{2}}$, probably via the established powerful influence on CBF and CDO_2_, acts as a key mechanism resulting in a mismatch between cerebral oxygen supply and demand that necessitates elevation in OEF to maintain neurological function. The following discussion considers the evidence, experimental limitations and the implications of these findings.

The OEF is normally defined as the difference in arterial and venous oxygen content to reflect the fraction of oxygen extracted by tissue as blood passes through the capillary bed. In the brain, it is known that OEF reflects a balance between CBF (blood supply) and cerebral metabolic rate of oxygen (CMRO_2_; oxygen consumption), which can be considered the driving variables of OEF. More precisely, oxygen supply is better represented by CDO_2_ and is a product of CBF and $C_{aO_{2}}$. Although $C_{aO_{2}}$ was measured and was well maintained or slightly elevated during exercise, it was not possible to measure CBF accurately during all the experimental conditions. Despite this, it is well established that changes in $\Delta P_{\mathrm{aC}O_{2}}$ exert a highly influential effect on mediating changes in CBF both at rest (Hoiland et al., 2019; Kety & Schmidt, 1948; Markwalder et al., 1984; Shapiro et al., 1966; Willie et al., 2012) and during exercise (Ogoh & Ainslie, 2009; Ogoh et al., 2008; Olin et al., 2011; Subudhi et al., 2011). Likewise, owing to the absence of CBF measurements, it was not possible to measure CMRO_2_. Although CMRO_2_ is reported to increase by 20%−30% during progressive exercise (Fisher et al., 2013; Rasmussen et al., 2010; Scheinberg et al., 1954; Smith & Ainslie, 2017; Smith et al., 2014), it is unknown how this might be altered during supramaximal exercise; however, as discussed next, given that CDO_2_ is markedly reduced at this time point, OEF is likely to be elevated in order to support metabolism.

### 4.1 Alterations in OEF during exercise are mediated by $\Delta P_{\mathrm{aC}O_{2}}$

From moderate to maximal exercise, OEF is maintained owing to the preservation of CDO_2_ via apt changes in CBF and $C_{aO_{2}}$ (Smith & Ainslie, 2017; Smith et al., 2014). Smith et al. (2014) demonstrate this relationship, whereby a peak in CBF at 60% of maximal workload (*W*_max_), corresponding to the ventilatory threshold, declines progressively thereafter to near baseline at maximal effort. The decline in CBF towards baseline is coupled with gradual but small increases in $C_{aO_{2}}$ at each stage (60%, 80% and 100% *W*_max_) and, therefore, CDO_2_ is maintained between 60% and 100% of *W*_max_. Consistent with these findings, in the present study, the OEF remained remarkably stable across SUB (baseline vs. submaximal: 42% ± 8% vs. 42% ± 6%) and even MAX (baseline vs. maximal: 44% ± 6% vs. 47% ± 6%) exercise. In contrast, supramaximal exercise elicited a marked ∼16% (absolute) increase in OEF from baseline (31% ± 4% vs. 47% ± 4%, respectively). Although CDO_2_ was not measured in the present study, it is likely that reductions in CBF, hence CDO_2_, contributed to the increased OEF in the brain with supramaximal efforts. This need for higher extraction during HIS is brought on by the hypocapnia that occurs with hyperventilation at such intensities. For example, it was recently reported that there is a striking mismatch between lower CDO_2_ and higher tissue metabolic requirements with hypocapnia (e.g., 5.1 mL/min reduction in CDO_2_ and 0.5 mL/min increase in CMRO_2_ per millimetre of mercury reduction in $\Delta P_{\mathrm{aC}O_{2}}$; Caldwell et al., 2024). Although OEF is statistically unaltered with SUB and MAX exercise, the variable is still closely correlated with the changes in $\Delta P_{\mathrm{aC}O_{2}}$, indicating that the relationship between $\Delta P_{\mathrm{aC}O_{2}}$ and OEF is maintained regardless of exercise per se (i.e., HYPO) and/or exercise intensity (i.e., SUB, MAX and HIS).

Acutely, CBF is regulated by the rapid diffusion of CO_2_ across the blood–brain barrier, altering local perivascular extracellular pH (Arieff et al., 1976; Caldwell et al., 2021; Lassen, 1968; Shapiro et al., 1966). Therefore, changes in $\Delta P_{\mathrm{aC}O_{2}}$, rather than arterial pH, modulate cerebrovascular resistance, delivery and, ultimately, OEF. In studies examining CBF modulation with changes in arterial pH (e.g., acidosis and alkalosis), effects on cerebrovascular resistance are seen only when, as a direct result of alterations in $\Delta P_{\mathrm{aC}O_{2}}$, CO_2_ diffuses across the vascular wall, altering perivascular extracellular pH (Arieff et al., 1976; Caldwell et al., 2022, 2021; Lassen, 1968; Schieve & Wilson, 1953). The significant exercise‐related drop in arterial pH across maximal and supramaximal conditions, consistent with metabolic acidosis, is one stimulus for mediating elevations in ventilation. During HIS, exercise‐induced metabolic acidosis occurs when the rate of ATP hydrolysis (H^+^ release) exceeds the maximal buffering capacity of the blood; the excess CO_2_ is then removed via hyperventilation (Nielsen et al., 2002; Robergs et al., 2004), and these alterations in $\Delta P_{\mathrm{aC}O_{2}}$ explain, in part, the CBF kinetics (Scheinberg et al., 1954; Smith et al., 2016) and resultant OEF response to exercise (Figure 2). In contrast, the resting hypocapnia trial incurred a profile consistent with that of respiratory alkalosis, whereby arterial pH was increased from pre‐ to postexercise (0.07 ± 0.01) and, although statistically significant, [HCO_3_^−^] was minimally changed (−1.4 ± 0.2 mmol/L) from baseline compared with MAX (−9.6 ± 0.1 mmol/L) and HIS (−18.2 ± 0.5 mmol/L). Despite an apparent pooled relationship between ΔpH_Arterial_ and ΔOEF (*p* *=* 0.030), the effect was not consistent across conditions. Interaction effects (omnibus test) did not reach significance (*p =* 0.078), and only the MAX [β = 86.71 (9.57, 163.85), *p =* 0.029) and HYPO [β = 206.9 (23.24, 390.57), *p =* 0.028] conditions demonstrated significant positive associations, whereas SUB (*p =* 0.941) and HIS (*p =* 0.571) demonstrated no effect. Notably, the CIs for these relationships were wide, indicating substantial uncertainty. Taken together, these findings suggest a weaker and inconsistent link between ΔpH_Arterial_ and ΔOEF compared with $\Delta P_{\mathrm{aC}O_{2}}$, which showed robust, consistently negative slopes across all conditions and narrower CIs, highlighting $\Delta P_{\mathrm{aC}O_{2}}$ as a stronger and more reliable predictor of OEF. Further studies are needed, perhaps with and without intravenous sodium bicarbonate infusion (Caldwell et al., 2021), with and without effective manipulation of $\Delta P_{\mathrm{aC}O_{2}}$, to determine the selective role in mediating changes in OEF during exercise.

### 4.2 Consideration of the Bohr effect

The Bohr effect (i.e., the influence of extracellular and intracellular H^+^, CO_2_ and temperature on haemoglobin–oxygen affinity) and the reciprocal Haldane effect (i.e., the influence of haemoglobin–oxygen saturation on H^+^ and CO_2_ binding) originate in the oxyhaemoglobin–deoxyhaemoglobin conformational change and allosteric interactions between oxygen and H^+^/CO_2_ binding sites (Malte et al., 2021). As the haemoglobin molecule shifts between the oxy and deoxy conformations in arterovenous gas transport, it delivers oxygen and binds CO_2_ and H^+^ in tissue capillaries (a pathway that is aided by the Bohr effect). The diffusion of oxygen from blood to the brain tissue is driven by the pressure (concentration) gradient (Hoiland et al., 2023). This gradient is determined, in part, by the quantity of oxygen released from haemoglobin upon deoxygenation. This deoxygenation is greatly affected by a number of factors, including $P_{CO_{2}}$, H^+^ and temperature.

In the present study, although $P_{CO_{2}}$ was matched between conditions, other effects of exercise (e.g., increased lactate, intra‐ and extracellular [H^+^], and temperature) will have altered the oxyhaemoglobin dissociation curve such that the magnitude of the Bohr effect will have differed considerably between HYPO and the higher‐intensity exercise conditions. Along with the effects of $P_{CO_{2}}$ on CBF, the differences in oxygen offloading at the tissues might well have had a role in shaping the present data and findings. For example, the Bohr effect is involved via the respiratory alkalosis induced by hyperventilation, and also via the intracellular pH change that results from modulation of red blood cell organic phosphate content (Jensen, 2004); such changes will ultimately lead to less oxygen unloading and a disturbed cerebral oxygen delivery. In contrast, in MAX and HIS conditions, although there is hypocapnia comparable with HYPO, there is also marked acidosis and elevations in temperature. These latter two influences promote oxygen offloading at the tissue level and are likely to offset the influence of hypocapnia on constraining unloading. Despite these competing influences on oxygen unloading, it is remarkable how comparable OEF is between hyperventilation‐induced hypocapnia per se (HYPO) and HIS. It seems possible that the mechanisms that determine OEF are independent of these overt changes in haemoglobin–oxygen affinity that are induced by exercise or hypocapnia alone. Our in vivo data cannot provide insight into these mechanisms, hence future research into the role of the oxygen dissociation (if any) in determining OEF is warranted; however, at least in in vivo human models, it will undoubtedly be challenging to disentangle the influence of CBF from dissociation curve effects.

### 4.3 Why does the brain extract so little oxygen?

Why might the brain, which is critically reliant on oxygen supply, extract so little oxygen compared with other organs, even at the highest workloads? For example, the coronary circulation extracts ∼70% of available oxygen at rest (Duncker & Bache, 2008; Jorgensen et al., 1977; Kitamura et al., 1972), whereas the brain extracts only ∼25%−40% (Lassen, 1959). At rest, skeletal muscle and the brain extract similar amounts relative to tissue mass; however, during exercise, the skeletal muscle can extract two to four times as much oxygen as the brain (Laughlin et al., 2011). Indeed, during heavy exercise, blood flow is redirected away from the renal, splanchnic and inactive muscle circulations to that of active skeletal muscle, thereby supporting metabolic demand (Joyner & Casey, 2015); at this point, the muscle extracts ∼90%−95% of the oxygen (Skattebo et al., 2020; Zheng et al., 2014). Even at the highest intensity exercise, cerebral OEF was increased by only ∼16% (absolute) from baseline to an absolute maximum of ∼47%.

In contrast to the muscle, the brain has a much lower bulk blood flow during exercise and reported limited ability to recruit capillaries, effectively fixing its diffusive surface area for oxygen extraction (Joyner & Casey, 2015; Poole & Musch, 2023; Poole et al., 2013). By tracking red blood cells across the cerebral capillary bed, constant capillary perfusion is reported (Kuschinsky & Paulson, 1992) and reiterated by comparable red blood cell transit times across a continuum of neural stimuli (Jespersen & Østergaard, 2012; Zoccoli et al., 1996). It is possible that the rate‐limiting step of cerebral OEF is a matter of supply, imposed by a fixed capillary network, rather than demand of cerebral tissue. Put another way, the OEF might not be a reflection of limited neuronal demand, as traditionally thought, but rather a result of limited blood flow at the capillary level (Herculano‐Houzel & Rothman, 2022). Taken together, the ability of the cardiovascular system to deliver oxygen to the brain (CBF), the anatomy of capillaries (diffusive capacity) and tissue oxygen tension homeostasis might be orchestrated such that the brain is unable to optimize its OEF. The precise direct and indirect mechanism(s) by which hypocapnia might mediate some elevations in OEF are unknown.

### 4.4 Experimental considerations

The present study has several considerations that might limit the interpretation of findings.

First, although using the gold‐standard Fick technique, the measurements of OEF in this work were on a whole‐brain level, which does not allow for regional interrogation. It is unknown, therefore, whether the association between OEF and $P_{CO_{2}}$ is maintained at the regional level. Currently, however, we know of no other region‐specific OEF techniques (e.g., MRI, PET with ^15^O‐labelled radiotracers) that would be applicable during whole‐body exercise, particularly at supramaximal levels.

Second, owing to practical constraints, the blood sampling during the SUB, MAX and HYPO trials is reflective of the physiological state during exercise and hypocapnia, respectively, whereas the HIS data reflect 2−3 min immediately after exercise. Nevertheless, regardless of the time point, the OEF data correspond to the measured $P_{CO_{2}}$ at that exact instance in the trial. Of note, the marked reduction in $P_{CO_{2}}$ post‐HIS trial indicates ongoing hyperventilation even 2−3 min after cessation of exercise. It is possible that 2−3 min post‐HIS, OEF compensation was not as high, because CDO_2_ appears to recover during this time in less intense exercise (Smith et al., 2014). Future studies are needed to determine the temporal changes in alveolar ventilation, metabolism, $P_{CO_{2}}$ and OEF to determine the kinetics of these relationships throughout exercise conditions and postexercise.

Third, owing to high levels of movement during intense exercise at or above maximal, or during marked hyperventilation‐induced hypocapnia, it is not feasible to obtain reliable CBF measures. However, as noted, it is well established that changes in $P_{CO_{2}}$ exert the most influential effect on mediating changes in CBF both at rest (Hoiland et al., 2019; Kety & Schmidt, 1948; Markwalder et al., 1984; Shapiro et al., 1966; Willie et al., 2012) and during exercise (Ogoh & Ainslie, 2009; Ogoh et al., 2008; Olin et al., 2011; Subudhi et al., 2011); therefore, we contend that any change in $P_{CO_{2}}$ is an excellent proxy for CBF.

Finally, the data represent a *post hoc*, exploratory assembly of experiments, collected for different purposes and with varying participant overlaps, which introduces inherent limitations (e.g., selection bias). For example, the same individuals participated in the SUB and MAX groups, and there was a small overlap of several participants in all conditions. It is possible that the results obtained are a matter of homogeneity of the participants rather than true physiological differences elicited by the intervention; however, robust statistical analyses (linear mixed models) that are able to account for repeated measures suggest the latter.

### 4.5 Implications

It is known that OEF is a primary index of the homeostasis of oxygen demand and supply to the brain and can be a useful predictive biomarker in a number of brain diseases (Engle et al., 2024; Fan et al., 2020; Jiang, Lin, Liu, Sur, Xu, Hazel, Pottanat, Yasar et al., 2020). Although this study was conducted in young healthy individuals, in cases where OEF is compromised, such as in older adults or in individuals with vascular disease, marked changes in $P_{CO_{2}}$ (via its powerful influence on CBF) can have adverse consequences. For example, according to one meta‐analysis, which considered only PET‐ and MRI‐derived OEF, individuals with ischaemic cerebrovascular disease, inclusive of Moyamoya disease, have elevated OEF compared with healthy control subjects (Engle et al., 2024). Likewise, patients with symptomatic carotid or cerebral vessel disease with elevated OEF are at a higher risk of having a stroke compared with healthy control subjects (Engle et al., 2024). A prediction interval of OEF that accounted for heterogeneity in healthy individuals was estimated to be 32.4%–45.3% and also indicated that an OEF of >46.7% is predictive of future ischaemic events (Engle et al., 2024). The mean resting OEF in the SUB and MAX conditions was 42.0 ± 8.0 and 43.6 ± 5.5, respectively, which is at the upper end of this range, although differences in techniques should be considered (e.g., MRI vs. blood gas oximetry) (Engle et al., 2024). Likewise, several MRI‐ (Catchlove et al., 2018; Jiang, Lin, Liu, Sur, Xu, Hazel, Pottanat, Yasar et al., 2020; Lu et al., 2011; Peng et al., 2014) and PET‐based (Aanerud et al., 2012) studies have demonstrated that OEF is elevated with healthy ageing, an effect that might be related to limited oxygen supply (CBF) or, although less likely, changes in oxygen demand (CMRO_2_) associated with ageing. Beyond use as a biomarker of cerebrovascular risk or ageing, a sustained increase in OEF might be problematic owing to the inverse relationship with OEF and capillary oxygen tension, whereby reduced oxygen tension in the capillaries with rising OEF might further limit CBF, hence CDO_2_ to brain tissue (Buxton & Frank, 1997; Gjedde et al., 2005, 2010). On the contrary, reduced OEF is implicated in cognitive decline and amyloid burden, suggesting a role in age‐related neurological disorders, such as Alzheimer's disease (Jiang, Lin, Liu, Sur, Xu, Hazel, Pottanat, Darrow et al., 2020). In this context, older adults or those with cerebrovascular disease might struggle with oxygen homeostasis in the brain, exacerbated by high‐intensity exercise, environmental challenge or comorbidities affecting $P_{CO_{2}}$, which, in turn, impact CBF.

## 5 CONCLUSION

The hypothesis‐generating findings presented herein demonstrate that high‐intensity, supramaximal cycling induces a greater increase of OEF than that of other, less‐intense cycling exercise. Resting hyperventilation‐induced hypocapnia also yielded a similar increase in OEF to high‐intensity sprinting, indicating that exercise is non‐obligatory in this process. Future studies are needed to validate and extend these findings and to determine the mechanistic role of $P_{CO_{2}}$ in OEF. Understanding the limits of OEF might have important implications for pathology, especially when $P_{CO_{2}}$ (hence CBF) might be altered during environmental or exercise stress.

## AUTHOR CONTRIBUTIONS

Lillian M. Brewster, Travis Dylan Gibbons, Hannah Grace Caldwell and Philip N. Ainslie conceived and designed research; Lillian M. Brewster, Travis Dylan Gibbons, Hannah Grace Caldwell, Connor A. Howe, Jennifer S. Duffy, Andrew R. Steele, Justin A. Monteleone, Jay M. J. R. Carr, Jodie Lauren Koep, Tenasia D. R. Monaghan, David B. MacLeod and Philip N. Ainslie contributed to the acquisition, analysis or interpretation of data for the work; Lillian M. Brewster, Travis Dylan Gibbons, Hannah Grace Caldwell, Connor A. Howe, Jennifer S. Duffy, Andrew R. Steele, Justin A. Monteleone, Jay M. J. R. Carr, Jodie Lauren Koep, Tenasia D. R. Monaghan, David B. MacLeod and Philip N. Ainslie drafted the work or revised it critically for important intellectual content. All authors approved the final version of the manuscript and agree to be accountable for all aspects of the work in ensuring that questions related to the accuracy or integrity of any part of the work are appropriately investigated and resolved. All persons designated as authors qualify for authorship, and all those who qualify for authorship are listed.

## CONFLICT OF INTEREST

None declared.

## Supporting information



Supporting Information

Supporting Information

## Data Availability

The data that support the findings of this study are available from the corresponding author upon reasonable request.
