# Supplementary material for: Cerebral oxygen extraction across different exercise intensities: Role of arterial PCO2
Source: Exp Physiol. 2025 Dec 1;111(11):5205–18. doi: 10.1113/EP092724 (PMC13395019; doi:10.1113/EP092724)
Supplement: Supplementary file 1 [file EPH-111-5205-s001.docx]

Supplementary Material 2A. Detailed linear mixed model output for the change in blood gasses, oximetry, and metabolites in each condition (Table 3).

| Variable | Condition | emmean | SE | df | lower.CL | upper.CL |
| --- | --- | --- | --- | --- | --- | --- |
| ΔPaO_2_ (mmHg) | SUB | -0.8560081 | 2.40062881 | 41.9668691 | -5.7007865 | 3.98877032 |
| ΔPaO_2_ (mmHg) | MAX | 1.71577309 | 2.40062881 | 41.9668691 | -3.1290053 | 6.56055148 |
| ΔPaO_2_ (mmHg) | HIS | 26.9264253 | 2.53621506 | 41.9971596 | 21.8081258 | 32.0447248 |
| ΔPaO_2_ (mmHg) | HYPO | 1.29596897 | 2.52128663 | 41.9959586 | -3.7922079 | 6.38414588 |
| ΔPaCO_2_ (mmHg) | SUB | 1.35290437 | 0.78396875 | 42 | -0.2292086 | 2.93501737 |
| ΔPaCO_2_ (mmHg) | MAX | -6.2156263 | 0.78396875 | 42 | -7.7977392 | -4.6335133 |
| ΔPaCO_2_ (mmHg) | HIS | -12.881818 | 0.83035242 | 42 | -14.557537 | -11.206099 |
| ΔPaCO_2_ (mmHg) | HYPO | -9.2258731 | 0.82492038 | 42 | -10.89063 | -7.5611164 |
| ΔOEF (%) | SUB | -0.9407066 | 1.51523424 | 41.2277901 | -4.0002709 | 2.1188576 |
| ΔOEF (%) | MAX | 2.51663113 | 1.51523424 | 41.2277901 | -0.5429331 | 5.57619537 |
| ΔOEF (%) | HIS | 15.6059374 | 1.56718625 | 41.9999628 | 12.4432274 | 18.7686474 |
| ΔOEF (%) | HYPO | 17.6889713 | 1.57526122 | 41.7693317 | 14.5094453 | 20.8684973 |
| ΔSaO_2_ (%) | SUB | -0.3533279 | 0.30114747 | 41.9825863 | -0.9610755 | 0.25441981 |
| ΔSaO_2_ (%) | MAX | -1.1199945 | 0.30114747 | 41.9825863 | -1.7277422 | -0.5122469 |
| ΔSaO_2_ (%) | HIS | 0.25987089 | 0.31841457 | 41.9982934 | -0.3827165 | 0.90245828 |
| ΔSaO_2_ (%) | HYPO | 0.88789202 | 0.31645241 | 41.998087 | 0.24926435 | 1.5265197 |
| ΔPvO_2_ (mmHg) | SUB | 1.17132409 | 0.92114318 | 41.4739408 | -0.6883169 | 3.03096509 |
| ΔPvO_2_ (mmHg) | MAX | 2.93138743 | 0.92114318 | 41.4739408 | 1.07174643 | 4.79102844 |
| ΔPvO_2_ (mmHg) | HIS | 0.45608818 | 0.95886825 | 41.9973096 | -1.47899 | 2.39116632 |
| ΔPvO_2_ (mmHg) | HYPO | -10.835447 | 0.96032505 | 41.8656887 | -12.773646 | -8.8972487 |
| ΔPvCO_2_ (mmHg) | SUB | 2.12218493 | 0.50309029 | 42 | 1.10690762 | 3.13746224 |
| ΔPvCO_2_ (mmHg) | MAX | -2.3704863 | 0.50309029 | 42 | -3.3857636 | -1.355209 |
| ΔPvCO_2_ (mmHg) | HIS | -5.8545455 | 0.53285573 | 42 | -6.9298918 | -4.7791991 |
| ΔPvCO_2_ (mmHg) | HYPO | -3.9199097 | 0.52936987 | 42 | -4.9882213 | -2.851598 |
| ΔHb_a_ (g/dL) | SUB | 0.35471924 | 0.10206216 | 41.6503438 | 0.14869823 | 0.56074026 |
| ΔHb_a_ (g/dL) | MAX | 1.33805258 | 0.10206216 | 41.6503438 | 1.13203156 | 1.54407359 |
| ΔHb_a_ (g/dL) | HIS | 1.21854989 | 0.10674819 | 41.9939976 | 1.00312241 | 1.43397737 |
| ΔHb_a_ (g/dL) | HYPO | 0.00545963 | 0.10663325 | 41.9228242 | -0.2097467 | 0.22066598 |
| ΔHb_v_ (g/dL) | SUB | 0.40060324 | 0.10289212 | 41.7445849 | 0.19292088 | 0.60828559 |
| ΔHb_v_ (g/dL) | MAX | 1.28393657 | 0.10289212 | 41.7445849 | 1.07625421 | 1.49161893 |
| ΔHb_v_ (g/dL) | HIS | 1.24224971 | 0.10790133 | 41.9929343 | 1.02449493 | 1.4600045 |
| ΔHb_v_ (g/dL) | HYPO | 0.03683824 | 0.10763512 | 41.9490359 | -0.180386 | 0.25406252 |
| ΔHct_a_ (%) | SUB | 1.02302663 | 0.30005685 | 41.7367994 | 0.41737425 | 1.62867901 |
| ΔHct_a_ (%) | MAX | 4.0646933 | 0.30005685 | 41.7367994 | 3.45904091 | 4.67034568 |
| ΔHct_a_ (%) | HIS | 3.61901813 | 0.31459464 | 41.9929857 | 2.9841373 | 4.25389896 |
| ΔHct_a_ (%) | HYPO | -0.0216487 | 0.31385473 | 41.9469922 | -0.6550569 | 0.61175954 |
| ΔHct_v_ (%) | SUB | 1.19563603 | 0.52418602 | 40.9321736 | 0.13696761 | 2.25430445 |
| ΔHct_v_ (%) | MAX | 3.92896937 | 0.52418602 | 40.9321736 | 2.87030094 | 4.98763779 |
| ΔHct_v_ (%) | HIS | 3.89851035 | 0.53811396 | 41.9889271 | 2.81254393 | 4.98447677 |
| ΔHct_v_ (%) | HYPO | -0.7933247 | 0.54321631 | 41.6278939 | -1.88987 | 0.30322059 |
| ΔpH_a_ | SUB | -0.0113869 | 0.01081416 | 41.1306943 | -0.0332244 | 0.01045062 |
| ΔpH_a_ | MAX | -0.1395259 | 0.01081416 | 41.1306943 | -0.1613634 | -0.1176883 |
| ΔpH_a_ | HIS | -0.2791224 | 0.01115712 | 41.9987572 | -0.3016384 | -0.2566064 |
| ΔpH_a_ | HYPO | 0.07389198 | 0.01123058 | 41.7260126 | 0.05122334 | 0.09656062 |
| ΔpH_v_ | SUB | -0.0051367 | 0.0095079 | 42 | -0.0243244 | 0.01405099 |
| ΔpH_v_ | MAX | -0.1249489 | 0.0095079 | 42 | -0.1441366 | -0.1057612 |
| ΔpH_v_ | HIS | -0.2710652 | 0.01007043 | 42 | -0.2913881 | -0.2507422 |
| ΔpH_v_ | HYPO | 0.03511481 | 0.01000455 | 42 | 0.01492481 | 0.05530482 |
| ΔSvO_2_ (%) | SUB | 0.28531923 | 1.42210684 | 41.5896792 | -2.5854477 | 3.15608611 |
| ΔSvO_2_ (%) | MAX | -3.1896808 | 1.42210684 | 41.5896792 | -6.0604477 | -0.3189139 |
| ΔSvO_2_ (%) | HIS | -15.131551 | 1.48493994 | 41.9950443 | -18.128291 | -12.13481 |
| ΔSvO_2_ (%) | HYPO | -16.415349 | 1.48466645 | 41.9043262 | -19.41173 | -13.418968 |
| ΔCaO_2_ | SUB | 0.38103525 | 0.13259632 | 42 | 0.11344506 | 0.64862545 |
| ΔCaO_2_ | MAX | 1.51577993 | 0.13259632 | 42 | 1.24818973 | 1.78337013 |
| ΔCaO_2_ | HIS | 1.7421694 | 0.1404414 | 42 | 1.45874717 | 2.02559162 |
| ΔCaO_2_ | HYPO | 0.17333691 | 0.13952266 | 42 | -0.1082312 | 0.45490503 |
| ΔCvO_2_ | SUB | 0.40290829 | 0.28306846 | 41.4233929 | -0.1685827 | 0.97439924 |
| ΔCvO_2_ | MAX | 0.31638732 | 0.28306846 | 41.4233929 | -0.2551036 | 0.88787826 |
| ΔCvO_2_ | HIS | -1.9248908 | 0.29426849 | 41.9982422 | -2.5187494 | -1.3310323 |
| ΔCvO_2_ | HYPO | -3.0686702 | 0.29493483 | 41.8474682 | -3.6639371 | -2.4734033 |
| ΔGlucose_a_ (mmol/L) | SUB | -1.5869471 | 0.19942987 | 41.9238965 | -1.9894345 | -1.1844597 |
| ΔGlucose_a_ (mmol/L) | MAX | -0.0202805 | 0.19942987 | 41.9238965 | -0.4227679 | 0.38220694 |
| ΔGlucose_a_ (mmol/L) | HIS | 1.13159028 | 0.21032368 | 41.9950163 | 0.70713841 | 1.55604214 |
| ΔGlucose_a_ (mmol/L) | HYPO | 0.01393165 | 0.20923345 | 41.9890021 | -0.4083218 | 0.43618513 |
| ΔGlucose_v_ (mmol/L) | SUB | -1.5590678 | 0.20173907 | 41.8739164 | -1.96623 | -1.1519055 |
| ΔGlucose_v_ (mmol/L) | MAX | -0.0924011 | 0.20173907 | 41.8739164 | -0.4995633 | 0.31476113 |
| ΔGlucose_v_ (mmol/L) | HIS | 0.85668774 | 0.21239193 | 41.9936027 | 0.42806154 | 1.28531393 |
| ΔGlucose_v_ (mmol/L) | HYPO | -0.3044951 | 0.21145599 | 41.9793961 | -0.7312368 | 0.12224658 |
| ΔLactate_a_ (mmol/L) | SUB | -0.4385345 | 0.64210576 | 41.9146613 | -1.7344345 | 0.85736559 |
| ΔLactate_a_ (mmol/L) | MAX | 12.3781322 | 0.64210576 | 41.9146613 | 11.0822322 | 13.6740323 |
| ΔLactate_a_ (mmol/L) | HIS | 18.7916715 | 0.67695317 | 41.9946836 | 17.4255196 | 20.1578234 |
| ΔLactate_a_ (mmol/L) | HYPO | -0.0383821 | 0.67354282 | 41.9873361 | -1.3976587 | 1.32089445 |
| ΔLactate_v_ (mmol/L) | SUB | -0.3809214 | 0.61699697 | 41.9187799 | -1.6261432 | 0.86430033 |
| ΔLactate_v_ (mmol/L) | MAX | 10.8357452 | 0.61699697 | 41.9187799 | 9.59052345 | 12.080967 |
| ΔLactate_v_ (mmol/L) | HIS | 17.232753 | 0.6505784 | 41.9948274 | 15.9198279 | 18.5456782 |
| ΔLactate_v_ (mmol/L) | HYPO | -0.0047281 | 0.64725873 | 41.9880857 | -1.3109601 | 1.30150385 |
| ΔHCO_3_^-^_a_ (mmol/L) | SUB | 0.42504509 | 0.38448211 | 42 | -0.3508712 | 1.20096141 |
| ΔHCO_3_^-^_a_ (mmol/L) | MAX | -9.6074458 | 0.38448211 | 42 | -10.383362 | -8.8315295 |
| ΔHCO_3_^-^_a_ (mmol/L) | HIS | -18.257947 | 0.40723008 | 42 | -19.07977 | -17.436123 |
| ΔHCO_3_^-^_a_ (mmol/L) | HYPO | -1.42408 | 0.40456604 | 42 | -2.2405273 | -0.6076327 |
| ΔHCO_3_^-^_v_ (mmol/L) | SUB | 0.54387648 | 0.42453247 | 42 | -0.3128647 | 1.40061769 |
| ΔHCO_3_^-^_v_ (mmol/L) | MAX | -8.031603 | 0.42453247 | 42 | -8.8883442 | -7.1748618 |
| ΔHCO_3_^-^_v_ (mmol/L) | HIS | -16.315533 | 0.44965002 | 42 | -17.222964 | -15.408103 |
| ΔHCO_3_^-^_v_ (mmol/L) | HYPO | 0.10570214 | 0.44670848 | 42 | -0.7957921 | 1.00719635 |

Data from linear mixed models for each conditions. Emmean, estimated marginal mean; SE, standard error; df, degrees of freedom; lower.CL, lower confidence limit (95% confidence interval); upper.CL, upper confidence limit (95% confidence interval). Subscript “a” represents brachial/radial arterial, and “v” represents internal jugular venous data. Delta (Δ) refers to the difference between pre vs. post condition. PO_2_, partial pressure of oxygen; PCO_2_, partial pressure of carbon dioxide; HCO_3_^-^, bicarbonate; SO_2_, oxygen saturation; Hct, hematocrit; Hb, hemoglobin; OEF, cerebral oxygen extraction fraction. SUB, submaximal exercise; MAX, maximal exercise; HIS, high intensity sprinting; HYPO, hypocapnia (resting).

Supplementary Material 2B. Detailed pairwise comparisons from linear mixed models analyzing the change in blood gasses, oximetry, and metabolites between conditions (Table 3).

| Variable | contrast | estimate | SE | df | t.ratio | p.value | formatted_p |
| --- | --- | --- | --- | --- | --- | --- | --- |
| ΔPaO_2_ (mmHg) | SUB - MAX | -2.5717812 | 3.25482441 | 22.8675911 | -0.7901444 | 0.85816001 | p = 0.858 |
| ΔPaO_2_ (mmHg) | SUB - HIS | -27.782433 | 3.46536957 | 38.0265653 | -8.0171632 | 6.38E-09 | p < 0.0001 |
| ΔPaO_2_ (mmHg) | SUB - HYPO | -2.151977 | 3.48336141 | 40.9843175 | -0.6177875 | 0.92581731 | p = 0.926 |
| ΔPaO_2_ (mmHg) | MAX - HIS | -25.210652 | 3.46536957 | 38.0265653 | -7.2750256 | 6.16E-08 | p < 0.0001 |
| ΔPaO_2_ (mmHg) | MAX - HYPO | 0.41980412 | 3.48336141 | 40.9843175 | 0.12051696 | 0.9993636 | p = 0.999 |
| ΔPaO_2_ (mmHg) | HIS - HYPO | 25.6304563 | 3.51438462 | 33.1918322 | 7.29301403 | 1.29E-07 | p < 0.0001 |
| ΔPaCO_2_ (mmHg) | SUB - MAX | 7.56853063 | 1.09435773 | 23.399756 | 6.91595664 | 2.46E-06 | p < 0.0001 |
| ΔPaCO_2_ (mmHg) | SUB - HIS | 14.2347226 | 1.14229656 | 38.3152868 | 12.4614947 | 0 | p < 0.0001 |
| ΔPaCO_2_ (mmHg) | SUB - HYPO | 10.5787775 | 1.14229656 | 41.0139405 | 9.26097288 | 7.98E-11 | p < 0.0001 |
| ΔPaCO_2_ (mmHg) | MAX - HIS | 6.66619193 | 1.14229656 | 38.3152868 | 5.83578042 | 5.42E-06 | p < 0.0001 |
| ΔPaCO_2_ (mmHg) | MAX - HYPO | 3.01024687 | 1.14229656 | 41.0139405 | 2.63525863 | 0.05515616 | p = 0.0552 |
| ΔPaCO_2_ (mmHg) | HIS - HYPO | -3.6559451 | 1.16789745 | 33.9669422 | -3.1303648 | 0.01790867 | p = 0.0179 |
| ΔOEF (%) | SUB - MAX | -3.4573378 | 1.77802104 | 21.3654839 | -1.9444864 | 0.23998414 | p = 0.24 |
| ΔOEF (%) | SUB - HIS | -16.546644 | 2.06180812 | 35.92832 | -8.0253074 | 9.36E-09 | p < 0.0001 |
| ΔOEF (%) | SUB - HYPO | -18.629678 | 2.1307461 | 40.1530881 | -8.743265 | 4.61E-10 | p < 0.0001 |
| ΔOEF (%) | MAX - HIS | -13.089306 | 2.06180812 | 35.92832 | -6.3484599 | 1.41E-06 | p < 0.0001 |
| ΔOEF (%) | MAX - HYPO | -15.17234 | 2.1307461 | 40.1530881 | -7.1206701 | 7.38E-08 | p < 0.0001 |
| ΔOEF (%) | HIS - HYPO | -2.0830339 | 2.02127624 | 30.4361751 | -1.0305538 | 0.73305692 | p = 0.733 |
| ΔSaO_2_ (%) | SUB - MAX | 0.76666667 | 0.41166892 | 23.0046048 | 1.86233798 | 0.27140628 | p = 0.271 |
| ΔSaO_2_ (%) | SUB - HIS | -0.6131987 | 0.43590261 | 38.1119479 | -1.4067334 | 0.50302372 | p = 0.503 |
| ΔSaO_2_ (%) | SUB - HYPO | -1.2412199 | 0.4375174 | 40.9964312 | -2.8369612 | 0.03419068 | p = 0.0342 |
| ΔSaO_2_ (%) | MAX - HIS | -1.3798654 | 0.43590261 | 38.1119479 | -3.165536 | 0.01542475 | p = 0.0154 |
| ΔSaO_2_ (%) | MAX - HYPO | -2.0078865 | 0.4375174 | 40.9964312 | -4.5892725 | 0.00023584 | p = 0.000236 |
| ΔSaO_2_ (%) | HIS - HYPO | -0.6280211 | 0.44306155 | 33.3964486 | -1.417458 | 0.49764962 | p = 0.498 |
| ΔPvO_2_ (mmHg) | SUB - MAX | -1.7600633 | 1.123345 | 21.6508988 | -1.5668057 | 0.41739011 | p = 0.417 |
| ΔPvO_2_ (mmHg) | SUB - HIS | 0.71523591 | 1.27576264 | 36.5979294 | 0.560634 | 0.9430276 | p = 0.943 |
| ΔPvO_2_ (mmHg) | SUB - HYPO | 12.0067714 | 1.3084962 | 40.5057064 | 9.17600782 | 1.16E-10 | p < 0.0001 |
| ΔPvO_2_ (mmHg) | MAX - HIS | 2.47529925 | 1.27576264 | 36.5979294 | 1.94025062 | 0.22943061 | p = 0.229 |
| ΔPvO_2_ (mmHg) | MAX - HYPO | 13.7668347 | 1.3084962 | 40.5057064 | 10.5211117 | 2.92E-12 | p < 0.0001 |
| ΔPvO_2_ (mmHg) | HIS - HYPO | 11.2915355 | 1.26080474 | 31.0855253 | 8.95581619 | 2.40E-09 | p < 0.0001 |
| ΔPvCO_2_ (mmHg) | SUB - MAX | 4.49267124 | 0.70227384 | 23.399756 | 6.39732105 | 8.26E-06 | p < 0.0001 |
| ΔPvCO_2_ (mmHg) | SUB - HIS | 7.97673039 | 0.73303726 | 38.3152868 | 10.8817529 | 1.59E-12 | p < 0.0001 |
| ΔPvCO_2_ (mmHg) | SUB - HYPO | 6.04209459 | 0.73303726 | 41.0139405 | 8.24254768 | 1.83E-09 | p < 0.0001 |
| ΔPvCO_2_ (mmHg) | MAX - HIS | 3.48405915 | 0.73303726 | 38.3152868 | 4.75290865 | 0.00016012 | p = 0.00016 |
| ΔPvCO_2_ (mmHg) | MAX - HYPO | 1.54942335 | 0.73303726 | 41.0139405 | 2.1137034 | 0.16581206 | p = 0.166 |
| ΔPvCO_2_ (mmHg) | HIS - HYPO | -1.9346358 | 0.74946592 | 33.9669422 | -2.5813526 | 0.06532575 | p = 0.0653 |
| ΔHb_a_ (g/dL) | SUB - MAX | -0.9833333 | 0.1282414 | 21.9172837 | -7.6678309 | 6.94E-07 | p < 0.0001 |
| ΔHb_a_ (g/dL) | SUB - HIS | -0.8638306 | 0.14317126 | 37.0637242 | -6.033548 | 3.28E-06 | p < 0.0001 |
| ΔHb_a_ (g/dL) | SUB - HYPO | 0.34925962 | 0.14599206 | 40.7079792 | 2.39231924 | 0.09465528 | p = 0.0947 |
| ΔHb_a_ (g/dL) | MAX - HIS | 0.11950269 | 0.14317126 | 37.0637242 | 0.8346835 | 0.83762737 | p = 0.838 |
| ΔHb_a_ (g/dL) | MAX - HYPO | 1.33259295 | 0.14599206 | 40.7079792 | 9.12784529 | 1.28E-10 | p < 0.0001 |
| ΔHb_a_ (g/dL) | HIS - HYPO | 1.21309026 | 0.14244637 | 31.6149084 | 8.51611899 | 6.43E-09 | p < 0.0001 |
| ΔHb_v_ (g/dL) | SUB - MAX | -0.8833333 | 0.1315845 | 22.0975854 | -6.7130502 | 5.27E-06 | p < 0.0001 |
| ΔHb_v_ (g/dL) | SUB - HIS | -0.8416465 | 0.14537079 | 37.3155086 | -5.7896532 | 6.84E-06 | p < 0.0001 |
| ΔHb_v_ (g/dL) | SUB - HYPO | 0.363765 | 0.14773173 | 40.8007764 | 2.46233499 | 0.08137848 | p = 0.0814 |
| ΔHb_v_ (g/dL) | MAX - HIS | 0.04168686 | 0.14537079 | 37.3155086 | 0.28676228 | 0.99164312 | p = 0.992 |
| ΔHb_v_ (g/dL) | MAX - HYPO | 1.24709833 | 0.14773173 | 40.8007764 | 8.44164191 | 1.02E-09 | p < 0.0001 |
| ΔHb_v_ (g/dL) | HIS - HYPO | 1.20541147 | 0.14523881 | 31.944025 | 8.29951346 | 1.05E-08 | p < 0.0001 |
| ΔHct_a_ (%) | SUB - MAX | -3.0416667 | 0.38314527 | 22.0812142 | -7.9386774 | 3.74E-07 | p < 0.0001 |
| ΔHct_a_ (%) | SUB - HIS | -2.5959915 | 0.42367722 | 37.2944116 | -6.127286 | 2.39E-06 | p < 0.0001 |
| ΔHct_a_ (%) | SUB - HYPO | 1.0446753 | 0.43068415 | 40.7934853 | 2.42561819 | 0.08811621 | p = 0.0881 |
| ΔHct_a_ (%) | MAX - HIS | 0.44567517 | 0.42367722 | 37.2944116 | 1.05192146 | 0.72030263 | p = 0.72 |
| ΔHct_a_ (%) | MAX - HYPO | 4.08634197 | 0.43068415 | 40.7934853 | 9.48802503 | 4.28E-11 | p < 0.0001 |
| ΔHct_a_ (%) | HIS - HYPO | 3.6406668 | 0.42313737 | 31.91493 | 8.6039832 | 4.73E-09 | p < 0.0001 |
| ΔHct_v_ (%) | SUB - MAX | -2.7333333 | 0.58847062 | 21.0998219 | -4.6448085 | 0.00073761 | p = 0.000738 |
| ΔHct_v_ (%) | SUB - HIS | -2.7028743 | 0.69779546 | 35.0735299 | -3.8734478 | 0.00242246 | p = 0.00242 |
| ΔHct_v_ (%) | SUB - HYPO | 1.98896072 | 0.72737259 | 39.6074005 | 2.73444551 | 0.04417969 | p = 0.0442 |
| ΔHct_v_ (%) | MAX - HIS | 0.03045902 | 0.69779546 | 35.0735299 | 0.04365035 | 0.99996952 | p = 1 |
| ΔHct_v_ (%) | MAX - HYPO | 4.72229405 | 0.72737259 | 39.6074005 | 6.49226284 | 5.90E-07 | p < 0.0001 |
| ΔHct_v_ (%) | HIS - HYPO | 4.69183504 | 0.67829636 | 29.7150586 | 6.91708719 | 6.80E-07 | p < 0.0001 |
| ΔpH_a_ | SUB - MAX | 0.12813895 | 0.01250454 | 21.2706966 | 10.2473964 | 6.36E-09 | p < 0.0001 |
| ΔpH_a_ | SUB - HIS | 0.26773547 | 0.01461104 | 35.6538647 | 18.3241861 | 0 | p < 0.0001 |
| ΔpH_a_ | SUB - HYPO | -0.0852789 | 0.01514308 | 39.9891288 | -5.6315423 | 9.07E-06 | p < 0.0001 |
| ΔpH_a_ | MAX - HIS | 0.13959652 | 0.01461104 | 35.6538647 | 9.55417883 | 1.36E-10 | p < 0.0001 |
| ΔpH_a_ | MAX - HYPO | -0.2134178 | 0.01514308 | 39.9891288 | -14.093425 | 4.70E-13 | p < 0.0001 |
| ΔpH_a_ | HIS - HYPO | -0.3530144 | 0.01428223 | 30.1945352 | -24.717028 | 5.00E-14 | p < 0.0001 |
| ΔpH_v_ | SUB - MAX | 0.11981215 | 0.01327226 | 23.399756 | 9.02725889 | 2.53E-08 | p < 0.0001 |
| ΔpH_v_ | SUB - HIS | 0.26592846 | 0.01385366 | 38.3152868 | 19.1955387 | 0 | p < 0.0001 |
| ΔpH_v_ | SUB - HYPO | -0.0402515 | 0.01385366 | 41.0139405 | -2.9054802 | 0.02888799 | p = 0.0289 |
| ΔpH_v_ | MAX - HIS | 0.14611631 | 0.01385366 | 38.3152868 | 10.5471274 | 4.03E-12 | p < 0.0001 |
| ΔpH_v_ | MAX - HYPO | -0.1600637 | 0.01385366 | 41.0139405 | -11.553892 | 9.48E-13 | p < 0.0001 |
| ΔpH_v_ | HIS - HYPO | -0.30618 | 0.01416415 | 33.9669422 | -21.616554 | 0 | p < 0.0001 |
| ΔSvO_2_ (%) | SUB - MAX | 3.475 | 1.76800321 | 21.8174067 | 1.96549417 | 0.23134091 | p = 0.231 |
| ΔSvO_2_ (%) | SUB - HIS | 15.4168698 | 1.98606029 | 36.9037468 | 7.7625387 | 1.69E-08 | p < 0.0001 |
| ΔSvO_2_ (%) | SUB - HYPO | 16.7006679 | 2.02937202 | 40.6427891 | 8.22947582 | 2.04E-09 | p < 0.0001 |
| ΔSvO_2_ (%) | MAX - HIS | 11.9418698 | 1.98606029 | 36.9037468 | 6.01284357 | 3.55E-06 | p < 0.0001 |
| ΔSvO_2_ (%) | MAX - HYPO | 13.2256679 | 2.02937202 | 40.6427891 | 6.51712344 | 4.87E-07 | p < 0.0001 |
| ΔSvO_2_ (%) | HIS - HYPO | 1.2837981 | 1.97119639 | 31.4232792 | 0.65127864 | 0.91430966 | p = 0.914 |
| ΔCaO_2_ | SUB - MAX | -1.1347447 | 0.18509386 | 23.399756 | -6.1306446 | 1.56E-05 | p < 0.0001 |
| ΔCaO_2_ | SUB - HIS | -1.3611341 | 0.19320198 | 38.3152868 | -7.0451356 | 1.21E-07 | p < 0.0001 |
| ΔCaO_2_ | SUB - HYPO | 0.20769834 | 0.19320198 | 41.0139405 | 1.07503217 | 0.70648641 | p = 0.706 |
| ΔCaO_2_ | MAX - HIS | -0.2263895 | 0.19320198 | 38.3152868 | -1.1717761 | 0.64798567 | p = 0.648 |
| ΔCaO_2_ | MAX - HYPO | 1.34244302 | 0.19320198 | 41.0139405 | 6.94839162 | 1.15E-07 | p < 0.0001 |
| ΔCaO_2_ | HIS - HYPO | 1.56883249 | 0.19753198 | 33.9669422 | 7.9421697 | 1.77E-08 | p < 0.0001 |
| ΔCvO_2_ | SUB - MAX | 0.08652098 | 0.3424135 | 21.5858053 | 0.25267981 | 0.99416739 | p = 0.994 |
| ΔCvO_2_ | SUB - HIS | 2.32779914 | 0.39062932 | 36.4630597 | 5.95909993 | 4.38E-06 | p < 0.0001 |
| ΔCvO_2_ | SUB - HYPO | 3.4715785 | 0.40129001 | 40.4403115 | 8.65104647 | 5.76E-10 | p < 0.0001 |
| ΔCvO_2_ | MAX - HIS | 2.24127817 | 0.39062932 | 36.4630597 | 5.73760868 | 8.67E-06 | p < 0.0001 |
| ΔCvO_2_ | MAX - HYPO | 3.38505752 | 0.40129001 | 40.4403115 | 8.43543936 | 1.12E-09 | p < 0.0001 |
| ΔCvO_2_ | HIS - HYPO | 1.14377935 | 0.38537283 | 30.9461786 | 2.96798127 | 0.0278576 | p = 0.0279 |
| ΔGlucose_a_ (mmol/L) | SUB - MAX | -1.5666667 | 0.26612512 | 22.6258005 | -5.8869553 | 3.18E-05 | p < 0.0001 |
| ΔGlucose_a_ (mmol/L) | SUB - HIS | -2.7185374 | 0.28630393 | 37.8514143 | -9.4952851 | 8.72E-11 | p < 0.0001 |
| ΔGlucose_a_ (mmol/L) | SUB - HYPO | -1.6008788 | 0.28862953 | 40.9518235 | -5.546483 | 1.11E-05 | p < 0.0001 |
| ΔGlucose_a_ (mmol/L) | MAX - HIS | -1.1518707 | 0.28630393 | 37.8514143 | -4.0232447 | 0.00145296 | p = 0.00145 |
| ΔGlucose_a_ (mmol/L) | MAX - HYPO | -0.0342121 | 0.28862953 | 40.9518235 | -0.118533 | 0.99939439 | p = 0.999 |
| ΔGlucose_a_ (mmol/L) | HIS - HYPO | 1.11765862 | 0.28912293 | 32.8203131 | 3.86568659 | 0.00265843 | p = 0.00266 |
| ΔGlucose_v_ (mmol/L) | SUB - MAX | -1.4666667 | 0.26545597 | 22.4346022 | -5.5250844 | 7.75E-05 | p < 0.0001 |
| ΔGlucose_v_ (mmol/L) | SUB - HIS | -2.4157555 | 0.28815411 | 37.6857031 | -8.3835539 | 2.28E-09 | p < 0.0001 |
| ΔGlucose_v_ (mmol/L) | SUB - HYPO | -1.2545727 | 0.29125437 | 40.9127254 | -4.3074811 | 0.00056496 | p = 0.000565 |
| ΔGlucose_v_ (mmol/L) | MAX - HIS | -0.9490888 | 0.28815411 | 37.6857031 | -3.2936849 | 0.01110658 | p = 0.0111 |
| ΔGlucose_v_ (mmol/L) | MAX - HYPO | 0.212094 | 0.29125437 | 40.9127254 | 0.72820881 | 0.88522711 | p = 0.885 |
| ΔGlucose_v_ (mmol/L) | HIS - HYPO | 1.16118284 | 0.28993524 | 32.5151278 | 4.00497317 | 0.00183247 | p = 0.00183 |
| ΔLactate_a_ (mmol/L) | SUB - MAX | -12.816667 | 0.85442261 | 22.5856722 | -15.000383 | 1.85E-12 | p < 0.0001 |
| ΔLactate_a_ (mmol/L) | SUB - HIS | -19.230206 | 0.92088717 | 37.8188356 | -20.882261 | 0 | p < 0.0001 |
| ΔLactate_a_ (mmol/L) | SUB - HYPO | -0.4001523 | 0.9288539 | 40.9447415 | -0.4308022 | 0.97284484 | p = 0.973 |
| ΔLactate_a_ (mmol/L) | MAX - HIS | -6.4135393 | 0.92088717 | 37.8188356 | -6.9645223 | 1.66E-07 | p < 0.0001 |
| ΔLactate_a_ (mmol/L) | MAX - HYPO | 12.4165143 | 0.9288539 | 40.9447415 | 13.3675645 | 8.19E-13 | p < 0.0001 |
| ΔLactate_a_ (mmol/L) | HIS - HYPO | 18.8300536 | 0.92926459 | 32.7571823 | 20.2633931 | 8.73E-14 | p < 0.0001 |
| ΔLactate_v_ (mmol/L) | SUB - MAX | -11.216667 | 0.82203468 | 22.6032263 | -13.645004 | 1.24E-11 | p < 0.0001 |
| ΔLactate_v_ (mmol/L) | SUB - HIS | -17.613674 | 0.88526993 | 37.8332222 | -19.896389 | 0 | p < 0.0001 |
| ΔLactate_v_ (mmol/L) | SUB - HYPO | -0.3761933 | 0.89272255 | 40.9479068 | -0.4214 | 0.97450383 | p = 0.975 |
| ΔLactate_v_ (mmol/L) | MAX - HIS | -6.3970078 | 0.88526993 | 37.8332222 | -7.2260534 | 7.38E-08 | p < 0.0001 |
| ΔLactate_v_ (mmol/L) | MAX - HYPO | 10.8404733 | 0.89272255 | 40.9479068 | 12.1431606 | 8.29E-13 | p < 0.0001 |
| ΔLactate_v_ (mmol/L) | HIS - HYPO | 17.2374811 | 0.89361456 | 32.7848552 | 19.2896154 | 8.93E-14 | p < 0.0001 |
| ΔHCO_3_^-^_a_ (mmol/L) | SUB - MAX | 10.0324909 | 0.53670631 | 23.399756 | 18.6927016 | 2.35E-14 | p < 0.0001 |
| ΔHCO_3_^-^_a_ (mmol/L) | SUB - HIS | 18.6829917 | 0.56021697 | 38.3152868 | 33.3495641 | 0 | p < 0.0001 |
| ΔHCO_3_^-^_a_ (mmol/L) | SUB - HYPO | 1.8491251 | 0.56021697 | 41.0139405 | 3.30073026 | 0.01038101 | p = 0.0104 |
| ΔHCO_3_^-^_a_ (mmol/L) | MAX - HIS | 8.65050082 | 0.56021697 | 38.3152868 | 15.4413402 | 0 | p < 0.0001 |
| ΔHCO_3_^-^_a_ (mmol/L) | MAX - HYPO | -8.1833658 | 0.56021697 | 41.0139405 | -14.607494 | 8.27E-13 | p < 0.0001 |
| ΔHCO_3_^-^_a_ (mmol/L) | HIS - HYPO | -16.833867 | 0.57277242 | 33.9669422 | -29.390149 | 0 | p < 0.0001 |
| ΔHCO_3_^-^_v_ (mmol/L) | SUB - MAX | 8.57547946 | 0.59261341 | 23.399756 | 14.4706133 | 2.12E-12 | p < 0.0001 |
| ΔHCO_3_^-^_v_ (mmol/L) | SUB - HIS | 16.8594095 | 0.6185731 | 38.3152868 | 27.2553227 | 0 | p < 0.0001 |
| ΔHCO_3_^-^_v_ (mmol/L) | SUB - HYPO | 0.43817434 | 0.6185731 | 41.0139405 | 0.70836306 | 0.89320672 | p = 0.893 |
| ΔHCO_3_^-^_v_ (mmol/L) | MAX - HIS | 8.28393008 | 0.6185731 | 38.3152868 | 13.3919985 | 0 | p < 0.0001 |
| ΔHCO_3_^-^_v_ (mmol/L) | MAX - HYPO | -8.1373051 | 0.6185731 | 41.0139405 | -13.154961 | 8.31E-13 | p < 0.0001 |
| ΔHCO_3_^-^_v_ (mmol/L) | HIS - HYPO | -16.421235 | 0.63243642 | 33.9669422 | -25.965037 | 0 | p < 0.0001 |

Data from linear mixed models, pairwise comparisons comparing delta variables between conditions. Subscript “a” represents brachial/radial arterial, and “v” represents internal jugular venous data. Delta (Δ) refers to the difference between pre vs. post condition. PO_2_, partial pressure of oxygen; PCO_2_, partial pressure of carbon dioxide; HCO_3_^-^, bicarbonate; SO_2_, oxygen saturation; Hct, hematocrit; Hb, hemoglobin; OEF, cerebral oxygen extraction fraction. SUB, submaximal exercise; MAX, maximal exercise; HIS, high intensity sprinting; HYPO, hypocapnia (resting).
