# Supplementary material for: Cerebral oxygen extraction across different exercise intensities: Role of arterial PCO2
Source: Exp Physiol. 2025 Dec 1:10.1113/EP092724. Online ahead of print. doi: 10.1113/EP092724 (PMC13395019; doi:10.1113/EP092724)
Supplement: Supplementary file 1 — Supporting Information [file EPH-9999-0-s002.docx]

Supplementary Material 1. Detailed statistical analyses of blood gases, oximetry, and metabolites (Table 2)

| **Variable** | **Group** | **Time** | **Type** | **Test** | **Comparison1 (mean±SD)** | **Comparison2**  **(mean±SD)** | **t_statistic** | **p_value** | **Significance** |
| --- | --- | --- | --- | --- | --- | --- | --- | --- | --- |
| **pO_2_ (mmHg)** | SUB | PRE | Arterial vs Venous | art_vs_venous | 97.92 ± 5.62 | 32.20 ± 4.20 | 28.5843786 | 1.13E-11 | ‡ |
| **pO_2_ (mmHg)** | SUB | POST | Arterial vs Venous | art_vs_venous | 97.21 ± 3.54 | 33.19 ± 2.99 | 40.7016733 | 2.39E-13 | ‡ |
| **pO_2_ (mmHg)** | SUB | POST | Arterial | pre_vs_post | 97.92 ± 5.62 | 97.21 ± 3.54 | 0.52545937 | 0.609692 |  |
| **pO_2_ (mmHg)** | SUB | POST | Venous | pre_vs_post | 32.20 ± 4.20 | 33.19 ± 2.99 | -1.3954564 | 0.190409 |  |
| **pO_2_ (mmHg)** | SUB | POST | A–V diff | pre_vs_post | 65.72 ± 7.96 | 64.02 ± 5.45 | 0.98238082 | 0.347026 |  |
| **pO_2_ (mmHg)** | MAX | PRE | Arterial vs Venous | art_vs_venous | 98.42 ± 4.83 | 31.78 ± 2.65 | 46.5956612 | 5.45E-14 | ‡ |
| **pO_2_ (mmHg)** | MAX | POST | Arterial vs Venous | art_vs_venous | 100.28 ± 9.20 | 34.54 ± 4.67 | 19.3646057 | 7.55E-10 | ‡ |
| **pO_2_ (mmHg)** | MAX | POST | Arterial | pre_vs_post | 98.42 ± 4.83 | 100.28 ± 9.20 | -0.6503393 | 0.528822 |  |
| **pO_2_ (mmHg)** | MAX | POST | Venous | pre_vs_post | 31.78 ± 2.65 | 34.54 ± 4.67 | -2.5241185 | 0.028268 | * |
| **pO_2_ (mmHg)** | MAX | POST | A–V diff | pre_vs_post | 66.63 ± 4.95 | 65.74 ± 11.76 | 0.23942285 | 0.81518 |  |
| **pO_2_ (mmHg)** | HIS | PRE | Arterial vs Venous | art_vs_venous | 88.31 ± 9.65 | 35.64 ± 1.85 | 17.9339554 | 6.21E-09 | ‡ |
| **pO_2_ (mmHg)** | HIS | POST | Arterial vs Venous | art_vs_venous | 115.27 ± 5.12 | 35.95 ± 2.81 | 38.5263063 | 3.31E-12 | ‡ |
| **pO_2_ (mmHg)** | HIS | POST | Arterial | pre_vs_post | 88.31 ± 9.65 | 115.27 ± 5.12 | -7.8360109 | 1.41E-05 | ‡ |
| **pO_2_ (mmHg)** | HIS | POST | Venous | pre_vs_post | 35.64 ± 1.85 | 35.95 ± 2.81 | -0.3018653 | 0.768939 |  |
| **pO_2_ (mmHg)** | HIS | POST | A–V diff | pre_vs_post | 52.67 ± 9.74 | 79.33 ± 6.83 | -6.7352202 | 5.14E-05 | ‡ |
| **pO_2_ (mmHg)** | HYPO | PRE | Arterial vs Venous | art_vs_venous | 88.06 ± 7.05 | 40.02 ± 2.95 | 20.3550665 | 1.81E-09 | ‡ |
| **pO_2_ (mmHg)** | HYPO | POST | Arterial vs Venous | art_vs_venous | 89.47 ± 6.42 | 29.12 ± 1.93 | 29.970448 | 4E-11 | ‡ |
| **pO_2_ (mmHg)** | HYPO | POST | Arterial | pre_vs_post | 88.06 ± 7.05 | 89.47 ± 6.42 | -1.0301247 | 0.327223 |  |
| **pO_2_ (mmHg)** | HYPO | POST | Venous | pre_vs_post | 40.02 ± 2.95 | 29.12 ± 1.93 | 13.0097066 | 1.36E-07 | ‡ |
| **pO_2_ (mmHg)** | HYPO | POST | A–V diff | pre_vs_post | 48.05 ± 7.83 | 60.34 ± 6.68 | -9.0373056 | 3.99E-06 | ‡ |
| **pCO_2_ (mmHg)** | SUB | PRE | Arterial vs Venous | art_vs_venous | 38.74 ± 3.73 | 50.11 ± 3.88 | -12.969333 | 5.21E-08 | ‡ |
| **pCO_2_ (mmHg)** | SUB | POST | Arterial vs Venous | art_vs_venous | 40.10 ± 3.33 | 52.23 ± 3.66 | -15.990028 | 5.81E-09 | ‡ |
| **pCO_2_ (mmHg)** | SUB | POST | Arterial | pre_vs_post | 38.74 ± 3.73 | 40.10 ± 3.33 | -3.2968627 | 0.007118 | † |
| **pCO_2_ (mmHg)** | SUB | POST | Venous | pre_vs_post | 50.11 ± 3.88 | 52.23 ± 3.66 | -4.521943 | 0.000869 | ‡ |
| **pCO_2_ (mmHg)** | SUB | POST | A–V diff | pre_vs_post | -11.36 ± 3.03 | -12.13 ± 2.63 | 1.55986332 | 0.147082 |  |
| **pCO_2_ (mmHg)** | MAX | PRE | Arterial vs Venous | art_vs_venous | 39.17 ± 3.42 | 50.95 ± 3.75 | -18.795672 | 1.04E-09 | ‡ |
| **pCO_2_ (mmHg)** | MAX | POST | Arterial vs Venous | art_vs_venous | 32.96 ± 3.89 | 48.58 ± 3.85 | -19.82726 | 5.87E-10 | ‡ |
| **pCO_2_ (mmHg)** | MAX | POST | Arterial | pre_vs_post | 39.17 ± 3.42 | 32.96 ± 3.89 | 6.82547339 | 2.86E-05 | ‡ |
| **pCO_2_ (mmHg)** | MAX | POST | Venous | pre_vs_post | 50.95 ± 3.75 | 48.58 ± 3.85 | 6.63556821 | 3.68E-05 | ‡ |
| **pCO_2_ (mmHg)** | MAX | POST | A–V diff | pre_vs_post | -11.78 ± 2.17 | -15.62 ± 2.73 | 5.52686044 | 0.000179 | ‡ |
| **pCO_2_ (mmHg)** | HIS | PRE | Arterial vs Venous | art_vs_venous | 40.46 ± 2.70 | 49.60 ± 2.24 | -23.155207 | 5.1E-10 | ‡ |
| **pCO_2_ (mmHg)** | HIS | POST | Arterial vs Venous | art_vs_venous | 27.58 ± 3.11 | 43.75 ± 1.92 | -17.845254 | 6.52E-09 | ‡ |
| **pCO_2_ (mmHg)** | HIS | POST | Arterial | pre_vs_post | 40.46 ± 2.70 | 27.58 ± 3.11 | 11.6411416 | 3.88E-07 | ‡ |
| **pCO_2_ (mmHg)** | HIS | POST | Venous | pre_vs_post | 49.60 ± 2.24 | 43.75 ± 1.92 | 12.5215073 | 1.96E-07 | ‡ |
| **pCO_2_ (mmHg)** | HIS | POST | A–V diff | pre_vs_post | -9.14 ± 1.31 | -16.16 ± 3.00 | 6.79751233 | 4.76E-05 | ‡ |
| **pCO_2_ (mmHg)** | HYPO | PRE | Arterial vs Venous | art_vs_venous | 44.03 ± 0.88 | 50.86 ± 3.82 | -5.929823 | 0.000145 | ‡ |
| **pCO_2_ (mmHg)** | HYPO | POST | Arterial vs Venous | art_vs_venous | 34.80 ± 2.15 | 46.94 ± 5.00 | -10.706164 | 8.48E-07 | ‡ |
| **pCO_2_ (mmHg)** | HYPO | POST | Arterial | pre_vs_post | 44.03 ± 0.88 | 34.80 ± 2.15 | 16.2608636 | 1.61E-08 | ‡ |
| **pCO_2_ (mmHg)** | HYPO | POST | Venous | pre_vs_post | 50.86 ± 3.82 | 46.94 ± 5.00 | 5.57831445 | 0.000235 | ‡ |
| **pCO_2_ (mmHg)** | HYPO | POST | A–V diff | pre_vs_post | -6.84 ± 3.82 | -12.14 ± 3.76 | 7.76827724 | 1.52E-05 | ‡ |
| **pH** | SUB | PRE | Arterial vs Venous | art_vs_venous | 7.42 ± 0.02 | 7.36 ± 0.01 | 14.4433556 | 1.7E-08 | ‡ |
| **pH** | SUB | POST | Arterial vs Venous | art_vs_venous | 7.41 ± 0.01 | 7.35 ± 0.01 | 17.3571175 | 2.43E-09 | ‡ |
| **pH** | SUB | POST | Arterial | pre_vs_post | 7.42 ± 0.02 | 7.41 ± 0.01 | 1.68737701 | 0.119648 |  |
| **pH** | SUB | POST | Venous | pre_vs_post | 7.36 ± 0.01 | 7.35 ± 0.01 | 1.59956701 | 0.138 |  |
| **pH** | SUB | POST | A–V diff | pre_vs_post | 0.06 ± 0.02 | 0.06 ± 0.01 | 0.70751731 | 0.493963 |  |
| **pH** | MAX | PRE | Arterial vs Venous | art_vs_venous | 7.42 ± 0.01 | 7.36 ± 0.01 | 16.8839775 | 3.26E-09 | ‡ |
| **pH** | MAX | POST | Arterial vs Venous | art_vs_venous | 7.28 ± 0.04 | 7.23 ± 0.03 | 14.3519829 | 1.81E-08 | ‡ |
| **pH** | MAX | POST | Arterial | pre_vs_post | 7.42 ± 0.01 | 7.28 ± 0.04 | 12.7005237 | 6.48E-08 | ‡ |
| **pH** | MAX | POST | Venous | pre_vs_post | 7.36 ± 0.01 | 7.23 ± 0.03 | 13.7479045 | 2.84E-08 | ‡ |
| **pH** | MAX | POST | A–V diff | pre_vs_post | 0.06 ± 0.01 | 0.05 ± 0.01 | 3.8506027 | 0.002697 | † |
| **pH** | HIS | PRE | Arterial vs Venous | art_vs_venous | 7.52 ± 0.18 | 7.47 ± 0.17 | 16.1145544 | 1.75E-08 | ‡ |
| **pH** | HIS | POST | Arterial vs Venous | art_vs_venous | 7.24 ± 0.15 | 7.20 ± 0.14 | 12.507122 | 1.98E-07 | ‡ |
| **pH** | HIS | POST | Arterial | pre_vs_post | 7.52 ± 0.18 | 7.24 ± 0.15 | 15.2476937 | 2.99E-08 | ‡ |
| **pH** | HIS | POST | Venous | pre_vs_post | 7.47 ± 0.17 | 7.20 ± 0.14 | 16.2099935 | 1.66E-08 | ‡ |
| **pH** | HIS | POST | A–V diff | pre_vs_post | 0.05 ± 0.01 | 0.04 ± 0.01 | 1.63732393 | 0.132604 |  |
| **pH** | HYPO | PRE | Arterial vs Venous | art_vs_venous | 7.38 ± 0.01 | 7.35 ± 0.01 | 6.1397947 | 0.00011 | ‡ |
| **pH** | HYPO | POST | Arterial vs Venous | art_vs_venous | 7.45 ± 0.01 | 7.38 ± 0.01 | 32.9262433 | 1.58E-11 | ‡ |
| **pH** | HYPO | POST | Arterial | pre_vs_post | 7.38 ± 0.01 | 7.45 ± 0.01 | -14.553077 | 4.67E-08 | ‡ |
| **pH** | HYPO | POST | Venous | pre_vs_post | 7.35 ± 0.01 | 7.38 ± 0.01 | -9.9623313 | 1.65E-06 | ‡ |
| **pH** | HYPO | POST | A–V diff | pre_vs_post | 0.03 ± 0.02 | 0.07 ± 0.01 | -7.1470027 | 3.12E-05 | ‡ |
| **HCO3- (mmol/L)** | SUB | PRE | Arterial vs Venous | art_vs_venous | 24.77 ± 2.24 | 27.28 ± 2.58 | -7.077308 | 2.05E-05 | ‡ |
| **HCO3- (mmol/L)** | SUB | POST | Arterial vs Venous | art_vs_venous | 25.19 ± 2.18 | 27.82 ± 2.25 | -10.702944 | 3.73E-07 | ‡ |
| **HCO3- (mmol/L)** | SUB | POST | Arterial | pre_vs_post | 24.77 ± 2.24 | 25.19 ± 2.18 | -1.513761 | 0.158277 |  |
| **HCO3- (mmol/L)** | SUB | POST | Venous | pre_vs_post | 27.28 ± 2.58 | 27.82 ± 2.25 | -1.5054811 | 0.160364 |  |
| **HCO3- (mmol/L)** | SUB | POST | A–V diff | pre_vs_post | -2.51 ± 1.23 | -2.63 ± 0.85 | 0.6532374 | 0.527021 |  |
| **HCO3- (mmol/L)** | MAX | PRE | Arterial vs Venous | art_vs_venous | 24.66 ± 2.27 | 27.36 ± 2.22 | -14.215777 | 2E-08 | ‡ |
| **HCO3- (mmol/L)** | MAX | POST | Arterial vs Venous | art_vs_venous | 15.05 ± 2.36 | 19.33 ± 2.62 | -14.771511 | 1.34E-08 | ‡ |
| **HCO3- (mmol/L)** | MAX | POST | Arterial | pre_vs_post | 24.66 ± 2.27 | 15.05 ± 2.36 | 35.0628086 | 1.22E-12 | ‡ |
| **HCO3- (mmol/L)** | MAX | POST | Venous | pre_vs_post | 27.36 ± 2.22 | 19.33 ± 2.62 | 37.7473161 | 5.45E-13 | ‡ |
| **HCO3- (mmol/L)** | MAX | POST | A–V diff | pre_vs_post | -2.70 ± 0.66 | -4.27 ± 1.00 | 4.85079379 | 0.00051 | ‡ |
| **HCO3- (mmol/L)** | HIS | PRE | Arterial vs Venous | art_vs_venous | 28.53 ± 2.87 | 30.99 ± 3.47 | -9.7494175 | 2E-06 | ‡ |
| **HCO3- (mmol/L)** | HIS | POST | Arterial vs Venous | art_vs_venous | 10.27 ± 2.45 | 14.68 ± 2.84 | -13.841151 | 7.55E-08 | ‡ |
| **HCO3- (mmol/L)** | HIS | POST | Arterial | pre_vs_post | 28.53 ± 2.87 | 10.27 ± 2.45 | 26.8156873 | 1.2E-10 | ‡ |
| **HCO3- (mmol/L)** | HIS | POST | Venous | pre_vs_post | 30.99 ± 3.47 | 14.68 ± 2.84 | 26.0376416 | 1.61E-10 | ‡ |
| **HCO3- (mmol/L)** | HIS | POST | A–V diff | pre_vs_post | -2.47 ± 0.84 | -4.41 ± 1.06 | 8.2245346 | 9.23E-06 | ‡ |
| **HCO3- (mmol/L)** | HYPO | PRE | Arterial vs Venous | art_vs_venous | 25.49 ± 0.99 | 27.33 ± 1.79 | -5.3315065 | 0.000332 | ‡ |
| **HCO3- (mmol/L)** | HYPO | POST | Arterial vs Venous | art_vs_venous | 24.06 ± 1.17 | 27.43 ± 2.64 | -5.4403142 | 0.000285 | ‡ |
| **HCO3- (mmol/L)** | HYPO | POST | Arterial | pre_vs_post | 25.49 ± 0.99 | 24.06 ± 1.17 | 13.2121062 | 1.18E-07 | ‡ |
| **HCO3- (mmol/L)** | HYPO | POST | Venous | pre_vs_post | 27.33 ± 1.79 | 27.43 ± 2.64 | -0.2357916 | 0.818353 |  |
| **HCO3- (mmol/L)** | HYPO | POST | A–V diff | pre_vs_post | -1.84 ± 1.14 | -3.37 ± 2.05 | 3.85575122 | 0.003182 | † |
| **SO_2_ (%)** | SUB | PRE | Arterial vs Venous | art_vs_venous | 98.24 ± 0.56 | 57.67 ± 8.07 | 17.3464252 | 2.45E-09 | ‡ |
| **SO_2_ (%)** | SUB | POST | Arterial vs Venous | art_vs_venous | 97.91 ± 0.71 | 57.73 ± 6.07 | 22.3733266 | 1.6E-10 | ‡ |
| **SO_2_ (%)** | SUB | POST | Arterial | pre_vs_post | 98.24 ± 0.56 | 97.91 ± 0.71 | 1.927749 | 0.080092 |  |
| **SO_2_ (%)** | SUB | POST | Venous | pre_vs_post | 57.67 ± 8.07 | 57.73 ± 6.07 | -0.0492726 | 0.961585 |  |
| **SO_2_ (%)** | SUB | POST | A–V diff | pre_vs_post | 40.58 ± 8.10 | 40.17 ± 6.22 | 0.28049385 | 0.784304 |  |
| **SO_2_ (%)** | MAX | PRE | Arterial vs Venous | art_vs_venous | 98.14 ± 0.52 | 55.86 ± 5.53 | 26.126634 | 2.99E-11 | ‡ |
| **SO_2_ (%)** | MAX | POST | Arterial vs Venous | art_vs_venous | 97.04 ± 1.28 | 52.45 ± 6.14 | 22.1650515 | 1.77E-10 | ‡ |
| **SO_2_ (%)** | MAX | POST | Arterial | pre_vs_post | 98.14 ± 0.52 | 97.04 ± 1.28 | 3.4119495 | 0.005805 | † |
| **SO_2_ (%)** | MAX | POST | Venous | pre_vs_post | 55.86 ± 5.53 | 52.45 ± 6.14 | 2.38813309 | 0.03598 | * |
| **SO_2_ (%)** | MAX | POST | A–V diff | pre_vs_post | 42.28 ± 5.61 | 44.59 ± 6.97 | -1.384429 | 0.193667 |  |
| **SO_2_ (%)** | HIS | PRE | Arterial vs Venous | art_vs_venous | 97.65 ± 1.32 | 67.15 ± 3.71 | 24.7535759 | 2.65E-10 | ‡ |
| **SO_2_ (%)** | HIS | POST | Arterial vs Venous | art_vs_venous | 97.92 ± 0.49 | 51.81 ± 4.22 | 34.7390741 | 9.26E-12 | ‡ |
| **SO_2_ (%)** | HIS | POST | Arterial | pre_vs_post | 97.65 ± 1.32 | 97.92 ± 0.49 | -0.5627091 | 0.586026 |  |
| **SO_2_ (%)** | HIS | POST | Venous | pre_vs_post | 67.15 ± 3.71 | 51.81 ± 4.22 | 8.47255479 | 7.1E-06 | ‡ |
| **SO_2_ (%)** | HIS | POST | A–V diff | pre_vs_post | 30.50 ± 4.09 | 46.11 ± 4.40 | -7.9166297 | 1.29E-05 | ‡ |
| **SO_2_ (%)** | HYPO | PRE | Arterial vs Venous | art_vs_venous | 95.31 ± 0.89 | 71.10 ± 3.49 | 22.1359358 | 7.94E-10 | ‡ |
| **SO_2_ (%)** | HYPO | POST | Arterial vs Venous | art_vs_venous | 96.20 ± 0.54 | 54.61 ± 3.58 | 38.0119243 | 3.79E-12 | ‡ |
| **SO_2_ (%)** | HYPO | POST | Arterial | pre_vs_post | 95.31 ± 0.89 | 96.20 ± 0.54 | -5.6561476 | 0.000211 | ‡ |
| **SO_2_ (%)** | HYPO | POST | Venous | pre_vs_post | 71.10 ± 3.49 | 54.61 ± 3.58 | 15.4965775 | 2.56E-08 | ‡ |
| **SO_2_ (%)** | HYPO | POST | A–V diff | pre_vs_post | 24.21 ± 3.63 | 41.59 ± 3.63 | -16.005185 | 1.87E-08 | ‡ |
| **Hct (%)** | SUB | PRE | Arterial vs Venous | art_vs_venous | 42.96 ± 3.28 | 42.67 ± 3.22 | 0.81086214 | 0.434638 |  |
| **Hct (%)** | SUB | POST | Arterial vs Venous | art_vs_venous | 43.95 ± 3.53 | 43.89 ± 3.50 | 0.29086441 | 0.776566 |  |
| **Hct (%)** | SUB | POST | Arterial | pre_vs_post | 42.96 ± 3.28 | 43.95 ± 3.53 | -3.1021309 | 0.010066 | * |
| **Hct (%)** | SUB | POST | Venous | pre_vs_post | 42.67 ± 3.22 | 43.89 ± 3.50 | -3.3379902 | 0.006617 | † |
| **Hct (%)** | SUB | POST | A–V diff | pre_vs_post | 0.28 ± 1.21 | 0.06 ± 0.69 | 1.12901644 | 0.282911 |  |
| **Hct (%)** | MAX | PRE | Arterial vs Venous | art_vs_venous | 43.77 ± 3.35 | 43.68 ± 3.34 | 0.38215644 | 0.709625 |  |
| **Hct (%)** | MAX | POST | Arterial vs Venous | art_vs_venous | 47.80 ± 3.57 | 47.63 ± 3.86 | 0.95425155 | 0.360456 |  |
| **Hct (%)** | MAX | POST | Arterial | pre_vs_post | 43.77 ± 3.35 | 47.80 ± 3.57 | -18.279242 | 1.4E-09 | ‡ |
| **Hct (%)** | MAX | POST | Venous | pre_vs_post | 43.68 ± 3.34 | 47.63 ± 3.86 | -13.985238 | 2.38E-08 | ‡ |
| **Hct (%)** | MAX | POST | A–V diff | pre_vs_post | 0.08 ± 0.76 | 0.17 ± 0.61 | -0.3949482 | 0.700432 |  |
| **Hct (%)** | HIS | PRE | Arterial vs Venous | art_vs_venous | 42.18 ± 3.50 | 42.24 ± 3.51 | -0.5122408 | 0.619605 |  |
| **Hct (%)** | HIS | POST | Arterial vs Venous | art_vs_venous | 45.83 ± 4.09 | 46.05 ± 4.33 | -1.8952451 | 0.087304 |  |
| **Hct (%)** | HIS | POST | Arterial | pre_vs_post | 42.18 ± 3.50 | 45.83 ± 4.09 | -10.593056 | 9.35E-07 | ‡ |
| **Hct (%)** | HIS | POST | Venous | pre_vs_post | 42.24 ± 3.51 | 46.05 ± 4.33 | -9.1351849 | 3.62E-06 | ‡ |
| **Hct (%)** | HIS | POST | A–V diff | pre_vs_post | -0.05 ± 0.35 | -0.23 ± 0.40 | 1.14993231 | 0.276929 |  |
| **Hct (%)** | HYPO | PRE | Arterial vs Venous | art_vs_venous | 42.64 ± 2.64 | 42.95 ± 3.04 | -1.3650646 | 0.202155 |  |
| **Hct (%)** | HYPO | POST | Arterial vs Venous | art_vs_venous | 42.60 ± 3.14 | 42.22 ± 3.46 | 0.37060619 | 0.718661 |  |
| **Hct (%)** | HYPO | POST | Arterial | pre_vs_post | 42.64 ± 2.64 | 42.60 ± 3.14 | 0.11118835 | 0.913667 |  |
| **Hct (%)** | HYPO | POST | Venous | pre_vs_post | 42.95 ± 3.04 | 42.22 ± 3.46 | 0.79536473 | 0.44487 |  |
| **Hct (%)** | HYPO | POST | A–V diff | pre_vs_post | -0.32 ± 0.77 | 0.38 ± 3.42 | -0.6116353 | 0.554429 |  |
| **Hb (g/dL)** | SUB | PRE | Arterial vs Venous | art_vs_venous | 14.00 ± 1.07 | 13.93 ± 1.06 | 0.66039636 | 0.522588 |  |
| **Hb (g/dL)** | SUB | POST | Arterial vs Venous | art_vs_venous | 14.34 ± 1.16 | 14.32 ± 1.14 | 0.26138665 | 0.798624 |  |
| **Hb (g/dL)** | SUB | POST | Arterial | pre_vs_post | 14.00 ± 1.07 | 14.34 ± 1.16 | -3.1336828 | 0.009515 | † |
| **Hb (g/dL)** | SUB | POST | Venous | pre_vs_post | 13.93 ± 1.06 | 14.32 ± 1.14 | -3.3696942 | 0.006256 | † |
| **Hb (g/dL)** | SUB | POST | A–V diff | pre_vs_post | 0.08 ± 0.39 | 0.02 ± 0.22 | 0.87314159 | 0.401245 |  |
| **Hb (g/dL)** | MAX | PRE | Arterial vs Venous | art_vs_venous | 14.29 ± 1.10 | 14.27 ± 1.09 | 0.33791545 | 0.741788 |  |
| **Hb (g/dL)** | MAX | POST | Arterial vs Venous | art_vs_venous | 15.62 ± 1.17 | 15.55 ± 1.25 | 1.14605097 | 0.276094 |  |
| **Hb (g/dL)** | MAX | POST | Arterial | pre_vs_post | 14.29 ± 1.10 | 15.62 ± 1.17 | -17.433433 | 2.32E-09 | ‡ |
| **Hb (g/dL)** | MAX | POST | Venous | pre_vs_post | 14.27 ± 1.09 | 15.55 ± 1.25 | -13.829608 | 2.67E-08 | ‡ |
| **Hb (g/dL)** | MAX | POST | A–V diff | pre_vs_post | 0.03 ± 0.26 | 0.07 ± 0.20 | -0.613349 | 0.552117 |  |
| **Hb (g/dL)** | HIS | PRE | Arterial vs Venous | art_vs_venous | 13.75 ± 1.15 | 13.78 ± 1.14 | -1.1744404 | 0.267428 |  |
| **Hb (g/dL)** | HIS | POST | Arterial vs Venous | art_vs_venous | 14.97 ± 1.32 | 15.04 ± 1.41 | -1.6408253 | 0.131868 |  |
| **Hb (g/dL)** | HIS | POST | Arterial | pre_vs_post | 13.75 ± 1.15 | 14.97 ± 1.32 | -10.79482 | 7.85E-07 | ‡ |
| **Hb (g/dL)** | HIS | POST | Venous | pre_vs_post | 13.78 ± 1.14 | 15.04 ± 1.41 | -9.1959138 | 3.41E-06 | ‡ |
| **Hb (g/dL)** | HIS | POST | A–V diff | pre_vs_post | -0.04 ± 0.10 | -0.06 ± 0.13 | 0.58167505 | 0.573663 |  |
| **Hb (g/dL)** | HYPO | PRE | Arterial vs Venous | art_vs_venous | 13.91 ± 0.85 | 14.02 ± 1.00 | -1.3987572 | 0.192127 |  |
| **Hb (g/dL)** | HYPO | POST | Arterial vs Venous | art_vs_venous | 13.91 ± 1.03 | 14.06 ± 1.00 | -2.1151086 | 0.06052 |  |
| **Hb (g/dL)** | HYPO | POST | Arterial | pre_vs_post | 13.91 ± 0.85 | 13.91 ± 1.03 | -1.411E-15 | 1 |  |
| **Hb (g/dL)** | HYPO | POST | Venous | pre_vs_post | 14.02 ± 1.00 | 14.06 ± 1.00 | -1 | 0.340893 |  |
| **Hb (g/dL)** | HYPO | POST | A–V diff | pre_vs_post | -0.11 ± 0.26 | -0.15 ± 0.24 | 0.37839717 | 0.713045 |  |
| **O_2_ content** | SUB | PRE | Arterial vs Venous | art_vs_venous | 18.73 ± 1.44 | 10.85 ± 1.70 | 17.61585 | 2.08E-09 | ‡ |
| **O_2_ content** | SUB | POST | Arterial vs Venous | art_vs_venous | 19.11 ± 1.51 | 11.19 ± 1.58 | 22.3021104 | 1.66E-10 | ‡ |
| **O_2_ content** | SUB | POST | Arterial | pre_vs_post | 18.73 ± 1.44 | 19.11 ± 1.51 | -3.0394035 | 0.011259 | * |
| **O_2_ content** | SUB | POST | Venous | pre_vs_post | 10.85 ± 1.70 | 11.19 ± 1.58 | -1.0977449 | 0.295764 |  |
| **O_2_ content** | SUB | POST | A–V diff | pre_vs_post | 7.87 ± 1.55 | 7.91 ± 1.23 | -0.1692471 | 0.868675 |  |
| **O_2_ content** | MAX | PRE | Arterial vs Venous | art_vs_venous | 19.09 ± 1.42 | 10.78 ± 1.34 | 25.2957941 | 4.25E-11 | ‡ |
| **O_2_ content** | MAX | POST | Arterial vs Venous | art_vs_venous | 20.60 ± 1.48 | 11.03 ± 1.54 | 22.4414336 | 1.55E-10 | ‡ |
| **O_2_ content** | MAX | POST | Arterial | pre_vs_post | 19.09 ± 1.42 | 20.60 ± 1.48 | -13.104723 | 4.68E-08 | ‡ |
| **O_2_ content** | MAX | POST | Venous | pre_vs_post | 10.78 ± 1.34 | 11.03 ± 1.54 | -1.0050344 | 0.336477 |  |
| **O_2_ content** | MAX | POST | A–V diff | pre_vs_post | 8.31 ± 1.14 | 9.57 ± 1.48 | -3.8602442 | 0.002652 | † |
| **O_2_ content** | HIS | PRE | Arterial vs Venous | art_vs_venous | 18.24 ± 1.62 | 12.52 ± 1.47 | 25.4625937 | 2E-10 | ‡ |
| **O_2_ content** | HIS | POST | Arterial vs Venous | art_vs_venous | 19.98 ± 1.80 | 10.55 ± 1.37 | 28.103389 | 7.56E-11 | ‡ |
| **O_2_ content** | HIS | POST | Arterial | pre_vs_post | 18.24 ± 1.62 | 19.98 ± 1.80 | -12.045218 | 2.82E-07 | ‡ |
| **O_2_ content** | HIS | POST | Venous | pre_vs_post | 12.52 ± 1.47 | 10.55 ± 1.37 | 5.78743319 | 0.000176 | ‡ |
| **O_2_ content** | HIS | POST | A–V diff | pre_vs_post | 5.71 ± 0.74 | 9.43 ± 1.11 | -8.9164649 | 4.5E-06 | ‡ |
| **O_2_ content** | HYPO | PRE | Arterial vs Venous | art_vs_venous | 18.02 ± 1.05 | 13.47 ± 1.10 | 19.5430166 | 2.69E-09 | ‡ |
| **O_2_ content** | HYPO | POST | Arterial vs Venous | art_vs_venous | 18.19 ± 1.35 | 10.38 ± 1.05 | 29.5644125 | 4.58E-11 | ‡ |
| **O_2_ content** | HYPO | POST | Arterial | pre_vs_post | 18.02 ± 1.05 | 18.19 ± 1.35 | -1.1516908 | 0.276239 |  |
| **O_2_ content** | HYPO | POST | Venous | pre_vs_post | 13.47 ± 1.10 | 10.38 ± 1.05 | 13.8105959 | 7.71E-08 | ‡ |
| **O_2_ content** | HYPO | POST | A–V diff | pre_vs_post | 4.55 ± 0.77 | 7.81 ± 0.88 | -10.637434 | 9E-07 | ‡ |
| **Glucose (mmol/L)** | SUB | PRE | Arterial vs Venous | art_vs_venous | 6.78 ± 1.08 | 6.07 ± 1.12 | 15.1338885 | 1.04E-08 | ‡ |
| **Glucose (mmol/L)** | SUB | POST | Arterial vs Venous | art_vs_venous | 5.18 ± 0.54 | 4.50 ± 0.53 | 22.9850883 | 1.2E-10 | ‡ |
| **Glucose (mmol/L)** | SUB | POST | Arterial | pre_vs_post | 6.78 ± 1.08 | 5.18 ± 0.54 | 4.84785201 | 0.000513 | ‡ |
| **Glucose (mmol/L)** | SUB | POST | Venous | pre_vs_post | 6.07 ± 1.12 | 4.50 ± 0.53 | 4.76543238 | 0.000585 | ‡ |
| **Glucose (mmol/L)** | SUB | POST | A–V diff | pre_vs_post | 0.71 ± 0.16 | 0.68 ± 0.10 | 0.60892242 | 0.554943 |  |
| **Glucose (mmol/L)** | MAX | PRE | Arterial vs Venous | art_vs_venous | 5.49 ± 0.62 | 4.79 ± 0.60 | 18.9560896 | 9.49E-10 | ‡ |
| **Glucose (mmol/L)** | MAX | POST | Arterial vs Venous | art_vs_venous | 5.47 ± 0.52 | 4.69 ± 0.55 | 18.0814309 | 1.57E-09 | ‡ |
| **Glucose (mmol/L)** | MAX | POST | Arterial | pre_vs_post | 5.49 ± 0.62 | 5.47 ± 0.52 | 0.17036357 | 0.867817 |  |
| **Glucose (mmol/L)** | MAX | POST | Venous | pre_vs_post | 4.79 ± 0.60 | 4.69 ± 0.55 | 0.80837203 | 0.436011 |  |
| **Glucose (mmol/L)** | MAX | POST | A–V diff | pre_vs_post | 0.70 ± 0.13 | 0.77 ± 0.15 | -1.9148542 | 0.081864 |  |
| **Glucose (mmol/L)** | HIS | PRE | Arterial vs Venous | art_vs_venous | 5.25 ± 0.27 | 4.77 ± 0.26 | 21.2853122 | 1.17E-09 | ‡ |
| **Glucose (mmol/L)** | HIS | POST | Arterial vs Venous | art_vs_venous | 6.41 ± 0.63 | 5.65 ± 0.60 | 26.7881348 | 1.21E-10 | ‡ |
| **Glucose (mmol/L)** | HIS | POST | Arterial | pre_vs_post | 5.25 ± 0.27 | 6.41 ± 0.63 | -7.8371175 | 1.41E-05 | ‡ |
| **Glucose (mmol/L)** | HIS | POST | Venous | pre_vs_post | 4.77 ± 0.26 | 5.65 ± 0.60 | -5.7844767 | 0.000177 | ‡ |
| **Glucose (mmol/L)** | HIS | POST | A–V diff | pre_vs_post | 0.48 ± 0.08 | 0.75 ± 0.09 | -6.7082039 | 5.31E-05 | ‡ |
| **Glucose (mmol/L)** | HYPO | PRE | Arterial vs Venous | art_vs_venous | 5.33 ± 0.35 | 4.89 ± 0.34 | 14.0932853 | 6.36E-08 | ‡ |
| **Glucose (mmol/L)** | HYPO | POST | Arterial vs Venous | art_vs_venous | 5.36 ± 0.39 | 4.62 ± 0.58 | 6.68636275 | 5.46E-05 | ‡ |
| **Glucose (mmol/L)** | HYPO | POST | Arterial | pre_vs_post | 5.33 ± 0.35 | 5.36 ± 0.39 | -1.1744404 | 0.267428 |  |
| **Glucose (mmol/L)** | HYPO | POST | Venous | pre_vs_post | 4.89 ± 0.34 | 4.62 ± 0.58 | 2.58774585 | 0.027054 | * |
| **Glucose (mmol/L)** | HYPO | POST | A–V diff | pre_vs_post | 0.44 ± 0.10 | 0.75 ± 0.37 | -3.1063509 | 0.01113 | * |
| **Lactate (mmol/L)** | SUB | PRE | Arterial vs Venous | art_vs_venous | 1.09 ± 0.41 | 1.10 ± 0.35 | -0.3206302 | 0.754499 |  |
| **Lactate (mmol/L)** | SUB | POST | Arterial vs Venous | art_vs_venous | 0.67 ± 0.19 | 0.73 ± 0.18 | -4.6904158 | 0.00066 | ‡ |
| **Lactate (mmol/L)** | SUB | POST | Arterial | pre_vs_post | 1.09 ± 0.41 | 0.67 ± 0.19 | 3.50344563 | 0.00494 | † |
| **Lactate (mmol/L)** | SUB | POST | Venous | pre_vs_post | 1.10 ± 0.35 | 0.73 ± 0.18 | 3.36565886 | 0.0063 | † |
| **Lactate (mmol/L)** | SUB | POST | A–V diff | pre_vs_post | -0.01 ± 0.09 | -0.07 ± 0.05 | 2.24441154 | 0.046342 | * |
| **Lactate (mmol/L)** | MAX | POST | Arterial vs Venous | art_vs_venous | 13.07 ± 1.45 | 11.62 ± 1.25 | 12.3830157 | 8.42E-08 | ‡ |
| **Lactate (mmol/L)** | MAX | POST | Arterial | pre_vs_post | 0.68 ± 0.19 | 13.07 ± 1.45 | -28.28826 | 1.26E-11 | ‡ |
| **Lactate (mmol/L)** | MAX | POST | Venous | pre_vs_post | 0.78 ± 0.19 | 11.62 ± 1.25 | -28.545743 | 1.14E-11 | ‡ |
| **Lactate (mmol/L)** | MAX | POST | A–V diff | pre_vs_post | -0.10 ± 0.00 | 1.44 ± 0.40 | -13.241953 | 4.2E-08 | ‡ |
| **Lactate (mmol/L)** | HIS | PRE | Arterial vs Venous | art_vs_venous | 0.75 ± 0.15 | 0.79 ± 0.14 | -1.7888544 | 0.103921 |  |
| **Lactate (mmol/L)** | HIS | POST | Arterial vs Venous | art_vs_venous | 19.55 ± 4.23 | 18.02 ± 4.12 | 9.27624819 | 3.15E-06 | ‡ |
| **Lactate (mmol/L)** | HIS | POST | Arterial | pre_vs_post | 0.75 ± 0.15 | 19.55 ± 4.23 | -14.863582 | 3.82E-08 | ‡ |
| **Lactate (mmol/L)** | HIS | POST | Venous | pre_vs_post | 0.79 ± 0.14 | 18.02 ± 4.12 | -13.994027 | 6.8E-08 | ‡ |
| **Lactate (mmol/L)** | HIS | POST | A–V diff | pre_vs_post | -0.04 ± 0.07 | 1.53 ± 0.55 | -9.4596926 | 2.64E-06 | ‡ |
| **Lactate (mmol/L)** | HYPO | PRE | Arterial vs Venous | art_vs_venous | 0.46 ± 0.16 | 0.52 ± 0.16 | -2.6311741 | 0.025112 | * |
| **Lactate (mmol/L)** | HYPO | POST | Arterial vs Venous | art_vs_venous | 0.48 ± 0.17 | 0.57 ± 0.17 | -5.5901699 | 0.000231 | ‡ |
| **Lactate (mmol/L)** | HYPO | POST | Arterial | pre_vs_post | 0.46 ± 0.16 | 0.48 ± 0.17 | -1.490712 | 0.16689 |  |
| **Lactate (mmol/L)** | HYPO | POST | Venous | pre_vs_post | 0.52 ± 0.16 | 0.57 ± 0.17 | -1.7466675 | 0.111279 |  |
| **Lactate (mmol/L)** | HYPO | POST | A–V diff | pre_vs_post | -0.05 ± 0.07 | -0.09 ± 0.05 | 1.30465615 | 0.221235 |  |
| **OEF (%)** | SUB | POST | OEF | pre_vs_post | 42.04 ± 8.01 | 41.47 ± 6.25 | 0.40195945 | 0.695414 |  |
| **OEF (%)** | MAX | POST | OEF | pre_vs_post | 43.57 ± 5.49 | 46.46 ± 6.40 | -2.0194159 | 0.068485 |  |
| **OEF (%)** | HIS | POST | OEF | pre_vs_post | 31.42 ± 3.83 | 47.25 ± 4.30 | -8.4526548 | 7.25E-06 | ‡ |
| **OEF (%)** | HYPO | POST | OEF | pre_vs_post | 25.27 ± 4.05 | 42.95 ± 3.78 | -12.693038 | 1.72E-07 | ‡ |

PO_2_, partial pressure of oxygen; PCO_2_, partial pressure of carbon dioxide; HCO_3_^-^, bicarbonate; SO_2_, oxygen saturation; Hct, hematocrit; Hb, hemoglobin; OEF, cerebral oxygen extraction fraction. SUB, submaximal exercise; MAX, maximal exercise; HIS, high intensity sprinting; HYPO, hypocapnia (resting). ‡p < 0.001, †p<0.01, *p<0.05.
